# Spin‐Momentum Locking and Ultrafast Spin‐Charge Conversion in Ultrathin Epitaxial Bi_1 − *x*_Sb_*x*_ Topological Insulator

**DOI:** 10.1002/advs.202301124

**Published:** 2023-04-25

**Authors:** E. Rongione, L. Baringthon, D. She, G. Patriarche, R. Lebrun, A. Lemaître, M. Morassi, N. Reyren, M. Mičica, J. Mangeney, J. Tignon, F. Bertran, S. Dhillon, P. Le Févre, H. Jaffrès, J.‐M. George

**Affiliations:** ^1^ Université Paris‐Saclay CNRS, Thales Unité Mixte de Physique CNRS/Thales F‐91767 Palaiseau France; ^2^ Laboratoire de Physique de l'Ecole Normale Supérieure ENS Université PSL, CNRS Sorbonne Université Universitè Paris Cité F‐75005 Paris France; ^3^ Université Paris‐Saclay Synchrotron SOLEIL L'Orme des Merisiers, Départementale 128 Saint‐Aubin F‐91190 France; ^4^ Université Paris‐Saclay CNRS, Centre de Nanosciences et de Nanotechnologies Palaiseau F‐91120 France

**Keywords:** angle‐resolved photoemission spectroscopy, spin‐charge conversion, spin‐resolved angle‐resolved photoemission spectroscopy, surface states THz‐TDS, topological insulator

## Abstract

The helicity of three‐dimensional (3D) topological insulator surface states has drawn significant attention in spintronics owing to spin‐momentum locking where the carriers' spin is oriented perpendicular to their momentum. This property can provide an efficient method to convert charge currents into spin currents, and vice‐versa, through the Rashba–Edelstein effect. However, experimental signatures of these surface states to the spin‐charge conversion are extremely difficult to disentangle from bulk state contributions. Here, spin‐ and angle‐resolved photo‐emission spectroscopy, and time‐resolved THz emission spectroscopy are combined to categorically demonstrate that spin‐charge conversion arises mainly from the surface state in Bi_1 − *x*_Sb_*x*_ ultrathin films, down to few nanometers where confinement effects emerge. This large conversion efficiency is correlated, typically at the level of the bulk spin Hall effect from heavy metals, to the complex Fermi surface obtained from theoretical calculations of the inverse Rashba–Edelstein response. Both surface state robustness and sizeable conversion efficiency in epitaxial Bi_1 − *x*_Sb_*x*_ thin films bring new perspectives for ultra‐low power magnetic random‐access memories and broadband THz generation.

## Graphical abstract

Spin‐angle‐resolved photoemission spectroscopy and THz‐TDS are used to categorically demonstrate that spin‐charge conversion (SCC) arises at the surface of the Bi1‐xSbx topological insulator family ultrathin films owing to their spin‐momentum locking property. SCC occurs via the inverse Rashba–Edelstein effect down to few nanometers. This is shown to be correlated to the complex Fermi surface as obtained from advanced tight‐binding calculations.

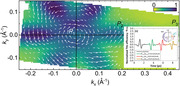

## 1 Introduction

The discovery of metallic quantum states at the surface of 3D topological insulators (TIs)^[^ ^1^, ^2^, ^3^ ^]^ has opened exciting new functionalities in spintronics owing to their topological protection and spin‐momentum locking (SML) properties.^[^ ^4^, ^5^ ^]^ Indeed, the combination of band inversion and time reversal symmetry (TRS) results in a peculiar spin texture in momentum space. Injecting a current in these states results therefore in an out of equilibrium spin density (also called spin‐accumulation) along the transverse direction.^[^ ^4^, ^5^, ^6^ ^]^ This is the Rashba–Edelstein effect (REE)^[^ ^7^ ^]^ which can be used to exert a spin‐orbit torque (SOT) onto the magnetization of an adjacent ferromagnet (FM).^[^ ^8^, ^9^ ^]^ The reciprocal phenomenon, by which a spin density produces an in‐plane transverse charge current, is called the inverse Rashba–Edelstein effect (IREE).^[^ ^6^, ^10^ ^]^

Importantly, the resulting spin‐charge conversion (SCC) efficiencies in topological surface states (TSS) combining strong spin‐orbit coupling (SOC) and SML is expected to be at least one order of magnitude larger compared to the spin Hall effect (SHE) of 5d heavy metals.^[^ ^11^, ^12^, ^13^ ^]^ SCC has been demonstrated in a range of Bi‐based TI compounds, including bismuth selenide Bi_2_Se_3_, bismuth telluride Bi_2_Te_3_, Bi_2_(Se,Te)_3_,^[^ ^14^ ^]^ or Bi_1 − *x*_Sb_*x*_ (BiSb).^[^ ^15^ ^]^ To benefit fully from IREE, the charge currents should be confined in the surface states and any current flowing through the bulk states should be avoided. The prerequisites are hence i) a sizeable bandgap typically larger than 0.2 to 0.3 eV, and ii) a perfect control of the Fermi level position, usually achieved by stoichiometry and/or strain engineering. In this respect, Bi_1 − *x*_Sb_*x*_ alloys, although displaying clear topological surface states,^[^ ^16^, ^17^, ^18^ ^]^ have been mostly neglected for spintronic applications as a result of their modest bulk bandgap (about 40 meV for *x* = 0.07) and relatively complex band structure. However, quantization effects in ultrathin films have shown to lead to much larger gap^[^ ^19^, ^20^ ^]^ while retaining their band inversion near the $\bar{M}$ point in the *x* = 0.07–0.3 composition range,^[^ ^21^ ^]^ unlike pure Bi.^[^ ^22^, ^23^, ^24^ ^]^ BiSb therefore has considerable potential as candidate for spintronics applications as well as for recently engineered efficient spintronic THz emitters.^[^ ^25^, ^26^, ^27^, ^28^, ^29^, ^30^, ^31^, ^32^ ^]^

In this letter, we report on our detailed investigation of the surface state SML properties of ultrathin (1 1 1)‐oriented Bi_1 − *x*_Sb_*x*_ epitaxial films. They exhibit a topological phase as recently confirmed by our angular‐resolved photo‐emission spectroscopy (ARPES) measurements.^[^ ^19^ ^]^ Here, we focus on spin‐resolved ARPES (SARPES) performed on ultrathin BiSb films and extract the in‐plane spin texture for the different electron and hole pockets characterizing the complex BiSb Fermi surface. Moreover, the SCC mediated by the BiSb surface states is probed at the sub‐picosecond timescale using an adjacent metallic Co layer acting as a spin injector. Unprecedentedly large SCC is measured with efficiencies beyond the level of carefully optimized Co/Pt systems. Our results also indicate that surface state related IREE is the mechanism responsible for SCC mechanism. Tight‐binding (TB) calculation and linear response theory account for our findings.

## 2 Spin‐Resolved ARPES

**Figure 1:**
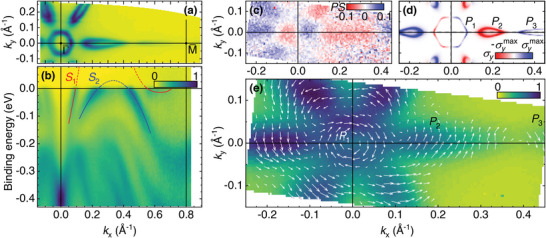
Spin‐resolved surface states of a 5‐nm thick Bi_0.85_Sb_0.15_ film grown on Si(1 1 1). a) High‐resolution ARPES map at the Fermi energy (integrated over 20 meV). b) ARPES energy dispersion along the $\bar{\Gamma }\bar{M}$ direction (*k*_*x*_ direction). As a guide to the eye, red and blue lines underline the *S*_1_ and *S*_2_ surface states. All ARPES measurements were performed at 20 K. The color bar represents the density of states in arb. units. c) σ_*y*_ polarization DOS measured at the Fermi level where the color bar is proportional to the spin‐polarization with scale extrema of *P* · *S* = ±0.1 (*T* = 300 K) and d) corresponding TB modeling of the s‐DOS projected on the first BL. The calculations are made at *T* = 0. The color bar indicates the spin polarization σ_*y*_ between −$\sigma_{y}^{\max}≃-0.7$ and $\sigma_{y}^{\max}≃+0.7$. e) Color map representing the measured ARPES intensity (arb. units, proportional to the DOS at the Fermi level) close to Fermi energy integrated on 25 meV with arrows representing the measured spin polarization direction and amplitude (*T* = 300 K). The different electron and hole pockets labeled *P*_1_, *P*_2_, and *P*_3_ in (d) are easily identified in the experimental measurements. The value of the colorbar indicates the full range (0–1) between 0 and the maximum value of *P* (0.5 ± 0.2).

SARPES is the method of choice to probe the spin‐textured Fermi contour of TI surfaces. Ultrathin epitaxial Bi_1 − *x*_Sb_*x*_ films with *x* = 0.07, 0.1, 0.15, 0.21, 0.3, 0.4, and thicknesses down to 2.5 nm were grown by molecular beam epitaxy (MBE) on clean 7 × 7 reconstructed Si(1 1 1) surfaces. They all exhibit a non‐trivial topological phase. Details of the growth^[^ ^33^ ^]^ are quickly recalled in Experimental Section where scanning transmission electron microscopy (STEM) and energy‐dispersive X‐ray spectroscopy (EDX) characterizations are also discussed. We focus first on the 5 nm thick Bi_0.85_Sb_0.15_ sample. **Figure** **1**a shows its experimental Fermi surface within the 2D surface Brillouin zone, as measured by ARPES. It is composed of three pockets labeled *P*_1_ (the hexagonal electron pocket surrounding $\bar{\Gamma }$), and two elongated *P*_2_ hole and *P*_3_ electron pockets along each of the six equivalent $\bar{\Gamma }\bar{M}$ directions (one has been chosen as the *k*_*x*_ axis). Figure 1b displays the energy dispersion along the $\bar{\Gamma }\bar{M}$ direction. The signature of two surface *S*_1_ and *S*_2_ states^[^ ^18^, ^19^ ^]^ are visible in the bandgap. The valence band state energy dispersion is also visible, from a series of confined states with energy splitting increasing with reducing thickness.^[^ ^19^ ^]^ The corresponding experimental SARPES polarization map for the σ_*y*_ spin‐polarization component is given in Figure 1c. For this experiment, the incident photon energy is 20 eV (5 meV resolution).

Owing to the electron analyzer movable entrance optics, the two in‐plane σ_*x*_ and σ_*y*_ spin‐DOS components were measured on a large part of the surface first Brillouin zone (see Experimental Section). On Figure 1e, these are represented as a vector field. The measured spin‐resolved DOS (s‐DOS) represents the DOS times the experimentally‐dependent factor *S* · *P* where *S* = 0.22 ± 0.07 is the spin‐detector Sherman function (see Experimental Section) and *P* is the k‐dependent electronic spin‐polarization. The spin(‐polarization) vector field is extracted accordingly and then superimposed over a color map yielding the sum of all the signals measured by the spin detector. For clarity, the vector fields were averaged over 0.02 × 0.02 Å^−2^ areas, corresponding to twenty data points. It is only displayed at positions where the DOS is significant. The experimental data reveals the helical spin texture of the inner *P*_1_ Fermi contour, the opposite spin polarization of the *P*_2_ hole pocket, and finally, the same spin chirality for the weaker *P*_3_ pocket. It yields a maximum spin‐polarization *P* = 0.5 ± 0.2 around the Fermi contours. We recover from experiments the symmetry property imposing the orthogonality between the spin direction and the vertical symmetry planes $\sigma_{V}^{\bar{\Gamma M}}$ containing the $\bar{\Gamma }\bar{M}$ lines. Such a property remains partly true concerning the in‐plane spin components for the $\bar{\Gamma }\bar{K}$ directions even if the corresponding vertical planes $\sigma_{V}^{\bar{\Gamma K}}$ do not represent perfect mirror planes. Such a lack of symmetry for $\sigma_{V}^{\bar{\Gamma K}}$ in hexagonal and rhombohedral structures leads to the so‐called warping term^[^ ^2^, ^34^, ^35^ ^]^ which may be responsible for the occurrence of a significant ±σ_*z*_ component. Note however that such a component, although generally leading to a reduction of the overall SCC efficiency,^[^ ^36^ ^]^ was not accessible in our SARPES experiments and will not be discussed further on.

We now compare the *S*_1_ and *S*_2_ surface state spin‐texture with a TB model. Calculations are implemented by considering relaxed bulk lattice parameters following the Vegard's law (refer to ref. [19] and Experimental Section). The electronic band structure and energy band dispersion along $\bar{M}\bar{\Gamma }\bar{M}$ are plotted in the Section S2, Supporting Information showing a good agreement with ARPES data. The σ_*y*_ s‐DOS at the Fermi surface, originating from *S*_1_ and *S*_2_, and projected on the top surface (first BL, *n* = 1) is displayed in Figure 1d. The color code represents the σ_*y*_‐DOS $N_{\text{DOS}}^{\sigma_{y}}$ projected onto the first BL. Around $\bar{\Gamma }$, positive (negative) values are observed for positive (negative) *k*_*x*_. The sign of this Rashba field is opposite around the $\bar{M}$ point (close to 0.8 Å^−1^). Two electron (*P*_1_, *P*_3_) and one hole (*P*_2_) pockets emerge, in very good agreement again with (S)ARPES data. By introducing the Rashba surface potential imposed by the surface symmetry breaking, we are able to reproduce the spin‐resolved map over the projected 2D‐Brillouin onto the first BiSb BL (see Experimental Section).

## 3 Ultrafast Spin‐Charge Conversion in Bi_1 − *x*_Sb_*x*_/Co Probed by THz‐TDS Emission Spectroscopy

With the clear demonstration of the spin resolved surface states, we now discuss the dynamical spin‐charge conversion in Bi_1 − *x*_Sb_*x*_ capped by a thin Co layer as a spin‐injector. THz emission spectroscopy in the time domain (THz‐TDS) has recently emerged as a powerful spectroscopic technique to investigate ultrafast SCC in materials with strong SOC^[^ ^25^ ^]^ and, in particular, in TI/FM structures.^[^ ^26^, ^27^, ^28^, ^32^ ^]^ The thin FM layer is excited from the top surface at normal incidence by a femtosecond laser pump leading to ultrafast demagnetization. This generates both spin density ${\hat{\mu }}_{s}$ and spin‐current $J_{s}$ diffusing toward the BiSb/Co interface owing to the two spin populations and mobilities introduced by the sp‐band spin‐splitting in Co.^[^ ^37^ ^]^ The spin is afterward converted into a transverse charge flow $J_{c}$ on sub‐picosecond timescales leading to a THz transient emission that is directly probed in the time‐domain (see refs. [ ^[^ ^25^, ^37^ ^]^] and Experimental Section). Two SCC mechanisms can contribute: the IREE from the surface states and the ISHE from the bulk. The THz electric field can be expressed as

(1) $$\left\{\begin{array}{cc} E_{\text{THz}}^{\text{ISHE}}\left(\theta \right) & \propto \frac{\partial J_{c}^{\text{ISHE}}}{\partial t}\propto i\omega \theta_{\text{SHE}}\;\left(J_{s}\times \frac{M(\theta )}{\left|M\right|}\right)\\ E_{\text{THz}}^{\text{IREE}}\left(\theta \right) & \propto \frac{\partial J_{c}^{\text{IREE}}}{\partial t}\propto i\omega \Lambda^{\text{IREE}}\;\left({\hat{\mu }}_{s}\times \frac{M(\theta )}{\left|M\right|}\right) \end{array}\right.$$

where **M**(θ) is the Co layer magnetization vector, ${\hat{\mu }}_{s}$ is the spin‐accumulation vector relaxing on *S*_1_ and *S*_2_ and ω is the frequency. **M**(θ) can be controlled by an external in‐plane magnetic field *B* at an angle θ from the *y* axis. In the above equation, $\theta_{\text{SHE}}=J_{c}/J_{s}$ is the spin Hall angle scaling the bulk ISHE‐mediated SCC, whereas Λ^IREE^ is the Rashba–Edelstein length scaling the IREE from the surface states (refer to Experimental Section and Section S5, Supporting Information).

**Figure 2:**
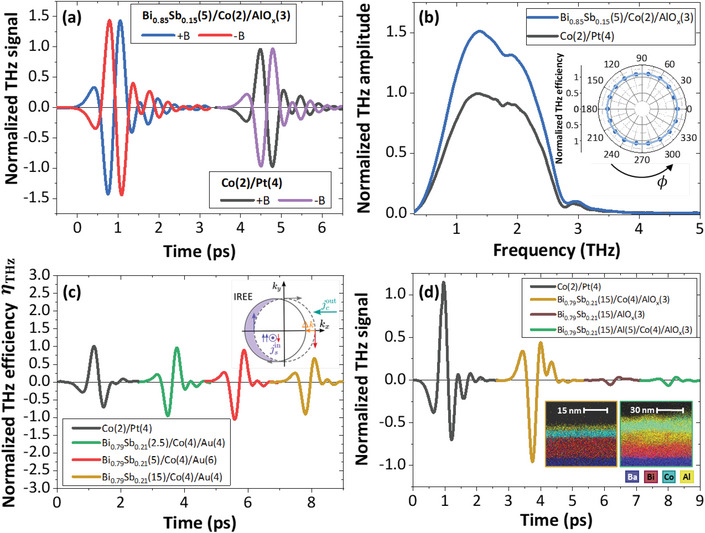
SCC and THz emission from Bi_1 − *x*_Sb_*x*_/Co bilayers acquired at 300 K. a) THz time‐trace from Bi_0.85_Sb_0.15_(5)/Co(2)/AlO_*x*_(3) for ±*B* compared to Co(2)/Pt(4) (grown on high resistivity Si substrates). THz phase reversal is a signature of SCC‐mediated THz emission. b) Spectral components of the THz emission from Bi_0.85_Sb_0.15_(5)/Co(2)/AlO_*x*_(3) and Co(2)/Pt(4) for +*B*. Inset) Normalized THz efficiency η_THz_ dependence on the azimuthal angle ϕ for Bi_0.85_Sb_0.15_(5)/Co(2)/AlO_*x*_(3). c) THz efficiency η_THz_ as a function of the Bi_0.79_Sb_0.21_ layer thickness (2.5, 5, and 15 nm) compared to Co(2)/Pt(4) (high resistivity Si substrates). d) THz signals from Bi_0.79_Sb_0.21_(15)/Al(5)/Co(4)/AlO_*x*_(3), Bi_0.79_Sb_0.21_(15)/AlO_*x*_(3), Bi_0.79_Sb_0.21_(15)/Co(4)/AlO_*x*_(3) on BaF_2_ (Section S3E, Supporting Information) and Co(2)/Pt(4). Time traces are shifted in time for clarity. Inset) Fluorescence map obtained from a TEM cross‐section for Bi_0.79_Sb_0.21_ (15)/Co(4)/AlOx(3) (brown frame) and Bi_0.79_Sb_0.21_(15)/Al(5)/Co(4)/AlOx(3) (green frame) grown on BaF_2_ for the elements and the color code given below the maps.

On **Figure** **2**a, we report the THz signal acquired at 300 K in reflection geometry (normal incidence, bilayer pumped from the Co side) from Bi_0.85_Sb_0.15_(5)/Co(2) sample (numbers in parenthesis are thicknesses in nm) at room temperature with a saturating in‐plane magnetic field *B* ≃ ±100 mT insuring that the magnetization follows the external field within much less than a degree. It is compared to the emission from our optimized Co(2)/Pt(4) metallic ISHE‐type sample. In both cases, we observe a short picosecond THz pulse with some minor oscillations within a 3 ps wide envelope. The THz signal phase changes sign when reversing the magnetic field, in full agreement with SCC‐mediated THz emission (Section S3A, Supporting Information). We note several differences: first, the amplitude from the BiSb/Co layer is 1.5 times larger than the Co/Pt revealing the large SCC efficiency in BiSb. For the same magnetic field orientation, the BiSb/Co THz signal phase is opposite to the one found in the Co/Pt sample (Figure 2a): this sign inversion is related to the inverted layer stacking of the FM and non magnetic layer, giving an opposite phase and indicating that Pt and BiSb share the same conversion sign.

We also report in Figure 2b the THz spectra (Fourier transform) obtained at 300 K from Co(2)/Pt(4) and Bi_1 − *x*_Sb_*x*_(5)/Co(2), demonstrating a relative power enhancement by a factor ≃ 2.3 for the Bi_1 − *x*_Sb_*x*_/Co sample (field enhancement by a factor ×1.5). The THz amplitude of Bi_1 − *x*_Sb_*x*_/Co is also shown to scale linearly with the pump fluence (Section S3B, Supporting Information), measured up to a few tens of µJ cm^−2^. The azimuthal angular dependence of the THz emission obtained by rotating the sample in the plane by an angle ϕ, while keeping fixed the magnetic field, is shown in the inset of Figure 2b. The emission is almost isotropic revealing a pure SCC phenomenon with no evidence of non‐linear optical effects such as shift or surge current contributions (e.g., photon drag, photogalvanic effects, etc.). This is in contrast with the recent report given by Park et al.^[^ ^31^ ^]^ where additional but small non‐magnetic contributions were observed. At normal incidence of the optical pump pulse, a pure isotropic THz response versus ϕ is expected from the linear response theory for both ISHE and IREE scenario (Section S5, Supporting Information) as discussed here. The role of the capping layer (metallic Au or oxidized Al, AlO_*x*_) has been carefully excluded by control experiments (Section S4B, Supporting Information). Two additional control samples were grown: i) a Bi_0.79_Sb_0.21_(15 nm) sample free of ferromagnetic Co and only capped with naturally oxidized AlO_*x*_(3 nm) almost emitting no THz radiation (Figure 2d) and ii) a sample with a 5 nm thick Al metallic spacer inserted between BiSb and Co. The Al insertion strongly reduces the THz emission. It may be explained either by a larger near‐infrared (NIR) and THz absorption in Al, or by interfacial spin‐loss or weakening of the surface SCC induced by the strong degradation of the interface quality. Indeed, TEM pictures displayed in the inset of Figure 2d reveal a strong intermixing of the Al interlayer that may induce a loss of an efficient spin‐injection and/or the alteration of the surface states (more details in Section S4A, Supporting Information).

To get a better insight into the SCC mechanism, the thickness dependence of the THz efficiency, η_THz_, acquired at 300 K is displayed in Figure 2c for the Bi_0.79_Sb_0.21_ series (2.5, 5, and 15 nm thick layers). η_THz_ represents the spin‐injection and conversion efficiencies and is obtained after having withdrawn the NIR and THz absorptions in the heterostructure following the procedure proposed in refs. [38, 39, 40] (Section S3C, Supporting Information). Strikingly, η_THz_ for BiSb remains constant for the whole thickness series where the BiSb bandgap widens dramatically by several hundreds of meV as the thickness is reduced, as calculated previously.^[^ ^19^ ^]^ Moreover, in agreement with our ARPES results,^[^ ^19^ ^]^ this demonstrates the absence of coupling between the top and bottom surface states^[^ ^41^ ^]^ down to 2.5 nm, unlike previously argued in the case of sputtered BiSb materials.^[^ ^29^ ^]^ The characteristic evanescence length is indeed ultrashort, typically 2 BL (0.8 nm) near $\bar{\Gamma }$ as confirmed by density functional theory^[^ ^42^ ^]^ for pure Bi and confirmed by our TB calculation (Section S5, Supporting Information). Such behavior is therefore more in favor of an interfacial origin of the SCC and thus strongly hints toward IREE from the surface states rather than ISHE from bulk.

Furthermore, when considering the possible contribution of bulk ISHE, BiSb thickness has to be compared with its spin diffusion length. It has been evaluated in bulk BiSb at λ_sf_ ≃ 8 nm by Sharma et al.,^[^ ^29^ ^]^ which is much larger than 2.5 nm. If bulk states were to contribute via ISHE, the spin current would therefore flow across the whole layer depth, and upper and lower interfaces would contribute similarly to the SCC but with an opposite sign. The net charge current would drop to zero, as $\tanh\left(\frac{t_{\text{TI}}}{\lambda_{\text{sf}}}\right)\tanh\left(\frac{t_{\text{TI}}}{2\lambda_{\text{sf}}}\right)\propto \frac{t_{\text{TI}}^{2}}{2\lambda_{\text{sf}}^{2}}$ ^[^ ^43^ ^]^ for small TI thicknesses *t*_TI_ when considering the multiple spin current reflections at the TI interfaces.^[^ ^40^ ^]^ This is in contrast with the thickness‐independent SCC observed here, in the ultrathin limit. We thus anticipate that the bulk states are hardly involved, and that the surface states at the FM/TI interface are mainly responsible for a net charge current through IREE. This conclusion is also supported by i) the large increase of the surface state DOS of BiSb at the interface compared to the DOS bulk states as the BiSb thickness increases (see Section S5, Supporting Information) and by ii) the assumption of the preservation of BiSb surface states in contact with Co, as it seems presently the case, providing that the proximity exchange remains here moderate and not exceeding few hundreds of meV (not shown).

**Figure 3:**
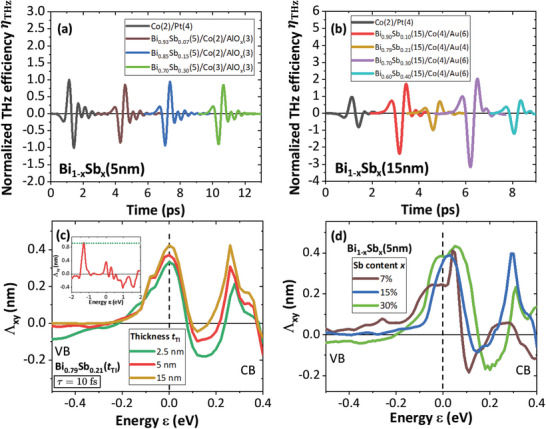
Sb content dependence of the THz efficiency η_THz_ acquired at 300 K and calculated IREE response for τ_s_ = 10 fs. a) η_THz_ from Bi_1 − *x*_Sb_*x*_(5)/Co(2‐3)/AlO_*x*_(3) with *x*=0.07, 0.15 and 0.3. b) η_THz_ from Bi_1 − *x*_Sb_*x*_(15)/Co(4)/Au(4‐6) with *x*=0.1, 0.21, 0.3, and 0.4. c,d) Values of the IREE length $\Lambda_{\mathrm{xy}}^{\text{IREE}}$ illustrating the conversion efficiency versus energy ε for c) Bi_0.79_Sb_0.21_ layers with thicknesses 2.5, 5, and 15 nm and d) Bi_1 − *x*_Sb_*x*_ (5 nm) for *x* = 0.07, 0.15, and 0.3. The inset presents the full IREE response outside the bandgap in the bulk valence and conduction bands for Bi_0.79_Sb_0.21_(5 nm). The calculations have been performed on for a spin‐relaxation time τ_s_ = τ = 10 fs after integration of the bands from to the top BL to the middle of the layer.

We now focus on the THz efficiency η_THz_ versus the Sb content *x*, plotted for Bi_1 − *x*_Sb_*x*_(5)/Co(2)/AlO_*x*_(3) in **Figure** **3**a and for Bi_1 − *x*_Sb_*x*_(15)/Co(4)/Au in Figure 3b (Section S3F, Supporting Information). In each series, a large η_THz_ is measured, comparable to that of Co/Pt with a maximum for *x* = 0.3 for the 15 nm series, that is, at the limit of the topological phase diagram.^[^ ^21^ ^]^ Importantly, η_THz_ remains similar for all the samples for the *t*_TI_ = 5 nm series (Figure 3a), highlighting again the prominent role of the surface states in the SCC. It also emphasizes the robustness of the IREE over a wide range of Sb content. Let us note that a sizeable THz signal persists outside the bulk topological window (*x* = 0.4 for 15 nm thickness in Figure 3b). This indicates that either i) the *S*_1_ Rashba state still partially contributes to SCC owing to its spin‐momentum locking property or ii) that the bulk states in heavily Sb‐doped BiSb start to contribute via ISHE, as theoretically calculated^[^ ^44^ ^]^ and demonstrated experimentally on torque experiments.^[^ ^9^ ^]^

## 4 SCC and IREE Tensor from the Linear Response Theory

We now compare the enhanced THz emission observed on ultrathin BiSb films to that obtained from the linear response theory described by the IREE response, namely $\Lambda_{\mathrm{xy}}^{\text{IREE}}$ matching with the so‐called inverse Edelstein length (see Section S5, Supporting Information and Experimental Section). We consider an extended formalism to the one recently developed to address the direct REE response as given in refs. [ ^[^ ^45^, ^46^, ^47^ ^]^]. We evaluate here the intraband response to an ongoing spin‐current relaxing onto the Fermi surface generating, via an out‐of‐equilibrium spin‐density, a charge current according to: $J_{c}^{x}=\Lambda_{\mathrm{xy}}^{\text{IREE}}\;J_{s,z}^{y}$ with $\Lambda_{\mathrm{xy}}^{\text{IREE}}=\frac{\sum_{n,k}\left(\sigma_{\mathrm{nk}}^{y}v_{\mathrm{nk}}^{x}\tau_{s}\frac{\partial f_{\mathrm{nk}}}{\partial \varepsilon }\right)}{\sum_{n,k}\left(\frac{\partial f_{\mathrm{nk}}}{\partial \varepsilon }\right)}$ where *y* is the direction of the spin injected with a flow along *z* whereas *x* is the direction of the in‐plane charge current. In the above expression, *n* is the band index, $v_{\mathrm{nk}}^{x}$ the corresponding band velocity along *x* and τ_s_ is the typical (spin) relaxation time assumed to be constant onto the Fermi surface. *f*_*n* **k**_ is the occupation number for the band *n* and wavevector **k** whereas $-\frac{\partial f_{nk}}{\partial \varepsilon }=N_{\text{DOS}}\left(\varepsilon ,k,n\right)$ represents the local DOS in the **k** space.

Calculations are performed on bare BiSb BLs free of any Co overlayer as investigated by SARPES. We show, on Figure 3c, the energy dependence of $\Lambda_{\mathrm{xy}}^{\text{IREE}}$ obtained for Bi_0.79_Sb_0.21_ of different film thicknesses: 2.5, 5, and 15 nm (see Methods). The Fermi level position corresponds to ε = 0. One observes that $\Lambda_{\mathrm{xy}}^{\text{IREE}}$ is largely enhanced in the bandgap region in the (−0.2–0.2) eV window where the *S*_2_ surface state and *S*_1_ TSS are located, however without being able to differentiate their individual contributions (see Figure S17, Supporting Information for the corresponding DOS in the Section S5, Supporting Information). Indeed the net SCC, as the IREE length $\Lambda_{\mathrm{xy}}^{\text{IREE}}\propto \langle \psi_{nk}|{\hat{v}}_{x}\tau_{s}|\psi_{nk}\rangle \langle \psi_{nk}|{\hat{\sigma }}_{y}\left|\psi_{nk}\right\rangle$, is connected to the product of their own helicity (clearly opposite between *S*_1_ and *S*_2_) times the carrier velocity. The sign of the carrier velocity is given by the sign of the derivative of the electronic dispersion (an example of calculation along the $\bar{\Gamma }\text{--}\bar{M}$ direction is given on Figure S18, Supporting Information in the Section S5, Supporting Information). In that sense, the sign of the spin‐helicity defining the spin‐momentum locking is not the only physical parameter to consider for calculating the resulting SCC current.

One may also observe that $\Lambda_{\mathrm{xy}}^{\text{IREE}}$ only slightly changes with the BiSb film thickness in this range (the maximum drop of $\Lambda_{\mathrm{xy}}^{\text{IREE}}$ near ε = ε_F_ is about −25% from 15 to 2.5 nm), in pretty close agreement with the experimental THz data (Figure 2c). Last, $\Lambda_{\mathrm{xy}}^{\text{IREE}}$ is maximum at the Fermi level, where it reaches values in the range of the equivalent SCC efficiency of Pt, product of the spin‐Hall angle by the spin‐diffusion length λ_sf_ as θ_SHE_ × λ_sf_ ≈ 0.2–0.3 nm for the same spin or momentum relaxation time (10 fs). Increasing the spin‐relaxation onto the surface states to 30 fs would yield $\Lambda_{\mathrm{xy}}^{\text{IREE}}\approx 1$ nm. $\Lambda_{\mathrm{xy}}^{\text{IREE}}$ is displayed in Figure 3c as a function of the Sb content *x* for the 5 nm series. Although one observes a slight increase of the SCC response from *x* = 0.1 to 0.3 in the gap window (still in line with the presence of the surface states, and possibly indicating a volume contribution), one may conclude that the SCC remains roughly constant at the vicinity of the Fermi energy (ε = ε_F_ = 0). This is observed experimentally from THz‐TDS measurements, and thus suggests an interfacial IREE nature of the SCC, at least for the thinner films. One cannot totally rule out a certain ISHE contribution arising from the bulk propagating bands associated to a very short spin‐diffusion length. Nonetheless, the strong impedance mismatch and subsequent spin‐backflow at Co/BiSb interface would be strongly in disfavor of such scenario.

## 5 Conclusions 

Although less investigated compared to other Bi‐based families because of its modest bandgap, BiSb ultrathin films still exhibit very robust surface states, as revealed by our spin‐resolved ARPES measurements. This is in part related to the confinement effects without being detrimental to the surface states. In particular, the presence of a topological surface state *S*_1_ in a wide Sb‐composition and thickness ranges is clearly observed, displaying helical spin texture at the Fermi surface with a specific opposite chirality between the electron pocket near the $\bar{\Gamma }$ point and the six hole pockets away from $\bar{\Gamma }$. Via ultrafast THz emission spectroscopy, we demonstrate that the complex spin‐texture gives rise to a very efficient spin‐charge conversion, resulting from the spin‐injection from a Co overlayer excited by an ultrashort laser pulse, mainly occurring via inverse Rashba–Edelstein effect (IREE) owing to the strong localization of the surface states. Our results address the role of spin‐textured hybridized Rashba‐like surface states offering unprecedented SCC efficiency despite the breaking of the TRS symmetry due to the local exchange interactions imposed by the magnetic contact. These results hold promise for efficient and integrated structures based on BiSb. Future investigations will concern the dynamics of the spin relaxation onto the BiSb surface states excited by ultrashort pulses.

## 6 Experimental Section

### MBE Growth of Bi_1 − *x*_Sb_*x*_:

The Bi_1 − *x*_Sb_*x*_ samples were grown by MBE. A Si(111) substrate was annealed in ultra‐high vacuum (UHV) at 1370 K in order to obtain a 7×7 surface reconstruction, observed by reflection high energy electron diffraction (RHEED). The Bi_1 − *x*_Sb_*x*_ alloy was grown by co‐deposition from two Knudsen cells. Each cell flux was calibrated prior to the deposition using a quartz microbalance and the relative deposition rates were directly used to estimate the Sb‐concentration *x*. RHEED measurements were performed throughout the deposition. Up to 5 nm, a 2D‐growth was observed; for thicker films, the evaporation was stopped at 7 nm for a 10 min intermediate annealing at 500 K before completing the film growth up to the targeted thickness. This procedure was further described in ref. [ ^[^ ^19^ ^]^]. BiSb films with thicknesses from 2.5 to 15 nm and with various compositions (0.03 < *x* < 0.3) were grown using this method and their crystallographic quality was demonstrated by RHEED, X‐ray‐diffraction, or STEM.^[^ ^19^ ^]^

All the samples were grown in the MBE chamber of the CASSIOPEE beamline installed on the SOLEIL synchrotron. After their elaboration, they can be transferred in UHV to an ARPES or a SARPES experiments where their electronic structure can be characterized. After photoemission measurements, some of the sample were transferred again into the MBE chamber for Co electron beam deposition. These bilayer systems were used for SCC measurements using THz emission spectroscopy.

### SEM–focused ion beam Milling and EDX:

Lamellae for STEM observation were prepared from the sample using focused ion beam (FIB) ion milling and thinning. Prior to FIB ion milling, the sample surface was coated with 50 nm of carbon to protect the surface from the platinum mask deposited used for the ion milling process. Ion milling and thinning were carried out in a FEI SCIOS dual‐beam FIB‐SEM. Initial etching was performed at 30 keV, and final polishing was performed at 5 keV. The lamellae were prepared following the two different zone axis (〈110〉 and 〈112〉) of the BaF_2_ substrate. All samples were observed in an aberration‐corrected FEI TITAN 200 TEM‐STEM operating at 200 keV. The convergence half‐angle of the probe was 17.6 mrad and the detection inner and outer half‐angles for HAADF‐STEM were 69 and 200 mrad, respectively. All micrographs where 2048 by 2048 pixels. The dwell time was 8 µs and the total acquisition time 41 s. EDX measurements were performed in the Titan microscope featuring the Chemistem system, that used a Bruker windowless Super‐X four‐quadrant detector and had a collection angle of 0.8 sr.

### (Spin‐) Angular‐Resolved Photoemission Spectroscopy:

The photoemission experiments were performed on the CASSIOPEE beamline installed on the SOLEIL storage ring (Saint‐Aubin, France). The beamline hosted two endstations. A high‐resolution ARPES endstation, which was used in this work for the measurement of the Fermi surface and of the band dispersion, using 20 eV incident photons with a linear horizontal polarization. It was equipped with a Scienta R4000 electron analyzer. The photon spot size on the sample was of the order of 50×50 µm^2^ and the overall kinetic energy resolution (taking into account both the photon energy and the electron kinetic energy resolutions) was of the order of 10 meV. The second endstation was a spin‐resolved ARPES experiment, where the beam size was ≈300×300 µm^2^. It was equipped with a MBS A1‐analyzer with a 2D detector for ARPES measurements. Close to this 2D detector, a 1×1 mm^2^ hole collected photoelectron with well‐defined kinetic energy and momentum. They were sent into a spin manipulator able to orient any spin component along the magnetization axis of a FERRUM VLEED spin‐detector, made of a Fe(100)‐p(1×1)O surface^[^ ^48^, ^49^ ^]^ deposited on a W‐substrate.

The spin polarization along the selected direction was proportional to the difference of the two signals collected for opposite magnetizations of the Fe‐oxide target. To reduce as much as possible the measurement asymmetries stemming from the instrument (i.e., not due to the spin polarization), four measurements per polarization direction were acquired, reversing both the ferrum magnetization direction, and the electron spin direction. This four measurements were combined into a geometrical average. The polarization was then determined by $P=S^{-1}\left(I_{+}^{\sigma }-I_{-}^{\sigma }\right)/\left(I_{+}^{\sigma }+I_{-}^{\sigma }\right)$ where the Sherman function *S* of the detector ranging between 0.15 and 0.3^[^ ^50^ ^]^ was estimated.

The 1×1 mm^2^ hole introduced an integration on both the kinetic energy and the wave vector. For the kinetic energy, it corresponded to 0.23% of the used pass energy (10 eV in this case), so ≈23 meV. Convoluted with the energy resolution of the analyzer (10 meV for this pass energy and an entrance slit of 400 µm), it gave an overall kinetic energy resolution of 25 meV. For the wave vector, the 1 mm aperture corresponded to an integration on 4% of the total (30°) angular range, which gives 1.2°. At 20 eV photon energy, for electrons at the Fermi level, this gave a *k*‐resolution of ≈0.048 Å^−1^. This explains the relatively broad featured of the SARPES in Figure 1c,e compared to Figure 1a.

The analyzer optics was movable and can collect electrons in a large 2D (30° × 30°) angular range. To map the spin texture at the Fermi level, the analyzer was set to the appropriate kinetic energy while the optics was moved by 0.2° step along two *X* and *Y* perpendicular directions. The two in‐plane spin components were measured at each step.

### THz Emission Spectroscopy:

Ultrafast NIR pulses (≃100 fs) centered at λ_NIR_=810 nm were derived from a Ti:Sapphire oscillator to photo‐excite the spin carriers directly from the front surface (Co side). Average powers of up to 600 mW were used with a repetition rate of 80 MHz (the energy per pulse is ≈3 nJ). The typical laser spot size on the sample was ≈200 µm × 200 µm. The optical pump was initially linearly polarized and irradiate the TI/FM heterostructure under normal incidence. The generated THz pulses were also collected from the front surface of the samples (i.e., reflection geometry) using a set of parabolic mirrors of 150 and 75 mm focal length to focus on the detection crystal. The samples were placed on a mount with a small magnetic field (≈100 mT) in the plane of the thin films. Both the sample orientation (angle ϕ) and the in‐plane magnetic field (angle θ) can be independently rotated in the sample plane. Standard electro‐optic sampling was used to detect the electric field of the THz pulses, using a 500 µm‐thick 〈1 1 0〉 ZnTe crystal. A chopper was placed at the focal point between the second and third parabolic mirror to modulate the THz beam at 6 kHz for heterodyne lock‐in detection. A mechanical delay line was used to sample the THz ultrafast pulse as a function of time. The THz propagation path was enclosed in a dry‐atmosphere purged chamber (typically <2% humidity) to reduce water absorption of the THz radiation.

### TB Calculations of Bi_1 − *x*_Sb_*x*_ Multilayers:

A TB model was developed in order to describe the Bi_1 − *x*_Sb_*x*_ electronic band structure as well as their surface topological properties.^[^ ^35^ ^]^ This approach was indeed well suited for TI and gave a fair description of the surface state spin texture in close agreement with the one derived from density functional theory developed for pure Bi surfaces.^[^ ^22^, ^23^, ^51^ ^]^ The rhombohedral A7 structure was described by two atoms per unit cell, forming then a BL of thickness of ≈0.4 nm. Bi_1 − *x*_Sb_*x*_ slabs were obtained by stacking the BL along the (1 1 1) direction (*z* axis) with two different plane‐to‐plane distances. The Hamiltonian was constructed on the basis of the work of ref. [35] using the generalization of the sp^3^ TB‐model Hamiltonian proposed for bulk Bi and Sb crystals,^[^ ^52^ ^]^ adapted to Bi_1 − *x*_Sb_*x*_ alloys^[^ ^16^ ^]^ and complemented by the introduction of additional surface potential terms when dealing with thin layers (treatment in slabs).^[^ ^35^, ^53^, ^54^ ^]^ In particular, the hopping parameters for the BiSb alloys were obtained by using the virtual crystal approximation (VCA) according to^[^ ^16^ ^]^

(2) $$V_{C}^{\text{BiSb}}=x\;V_{C}^{\text{Sb}}+\left(1-x^{2}\right)\;V_{C}^{\text{Bi}}$$

where *x* is the antimony content and $V_{C}^{\text{Sb}}$ and $V_{C}^{\text{Bi}}$ are the respective hopping parameters of Sb and Bi taken from ref. [52]. One notes ${\hat{\sigma }}_{\alpha }$ the spin index on each atom where α stands for the directional index. The hopping terms among the atomic orbitals were decomposed into inter‐ and intra‐BL hopping terms. The inter‐BL off‐diagonal hopping term between atoms (plane) 1 and atoms (plane) 2 consisted of the nearest‐neighbor coupling in the bulk BiSb Hamiltonian, whereas the intra‐BL hopping term consisted of two parts which represented respectively the third and second nearest neighbor contributions. The overall TB Hamiltonian was considered according to

(3) $$\hat{H}={\hat{H}}_{0}+{\hat{H}}_{\text{SO}}+{\hat{H}}_{\gamma }$$

where ${\hat{H}}_{0}=\Sigma_{i\mu }^{j\nu }\left|i\mu \right\rangle V_{i\mu }^{j\nu }\left\langle j\nu \right|$ represents the hopping Hamiltonian (*i*, *j* are the atomic positions, µ, ν are the orbitals), ${\hat{H}}_{\text{SO}}=\frac{\hbar }{4m^{2}c^{2}}\left(\vec{\nabla }V\left(r\right)\times \hat{p}\right)\cdot \hat{\sigma }$ the SOC term and ${\hat{H}}_{\gamma }$ the Rashba surface potential induced by the deformation of the surface orbitals due to the local electric field. Indeed, due to the symmetry breaking at the surface, a Rashba SOC term must be taken into account in the Hamiltonian at the two surface planes. Such an effect was modeled for the sp^3^ basis by using the approach of Ast and Gierz^[^ ^54^ ^]^ for ${\hat{H}}_{\gamma }$ considering two additional surface hopping terms γ_sp_ and γ_pp_ acting respectively between the s–p_*z*_ and p_*x*_–p_*z*_ (or p_*y*_–p_*z*_) surface orbitals. Thus the ${\hat{H}}_{\gamma }$ Hamiltonian term of the form was added

(4) $$\begin{array}{c} \hat{H_{\gamma }}=\left\{\begin{array}{cc} \gamma_{\text{sp}} & \left(i,i\right)\equiv \left(s,p_{z}\right)\\ \pm \gamma_{\text{sp}1} & \left(i,j\right)\equiv \left(s,p_{z}\right)\\ \pm \gamma_{\text{pp}}\cos\left(\theta \right) & \;\left(i,j\right)\equiv \left(p_{x},p_{z}\right)\\ \pm \gamma_{\text{pp}}\sin\left(\theta \right) & \;\left(i,j\right)\equiv \left(p_{y},p_{z}\right) \end{array}\right. \end{array}$$

where the + (−) sign corresponds to the uppermost (lowermost) atomic plane and θ is the angle between the direction joining the two atoms considered and the *x*‐direction. The authors then restrained themselves to the in‐plane surface hopping as for a pure 2D system. The best agreement with ARPES results was found by adding, as proposed in ref. [54], additional on‐site s–p_*z*_ coupling γ_sp_ = −0.2 eV, and surface hopping terms γ_sp1_ = 0.3 eV and γ_pp_ = −0.6 eV for *x* = 0.15 slightly departing from the values given for pure Bi, that is, γ_sp_ = 0.45 eV and γ_pp_ = −0.27 eV,^[^ ^35^ ^]^ with opposite sign for the top and bottom surfaces due to the opposite direction of the potential gradient. It was emphasized that this surface terms were required to correctly reproduce the surface state dispersion as observed by ARPES experiments. The size of the Hamiltonian $\hat{H}\left(k_{x},k_{y}\right)$ to diagonalize was 16*N* × 16*N* where *N* is the number of bilayers (BLs). Once the Green function of the multilayer system is defined as

(5) $$\hat{G}\left(\varepsilon ,k_{x},k_{y}\right)={\left[\varepsilon +i\delta -\hat{H}\left(k_{x},k_{y}\right)\right]}^{-1}$$

the partial density of state (DOS) $N_{\text{DOS}}\left(\varepsilon \right)$ versus the energy ε equals $N\left(n,\varepsilon \right)=-\left(1/\pi \right)\;\text{Im}\mathrm{Tr}[\hat{G}\left(\varepsilon ,n,k_{x},k_{y}\right)]$ whereas the spin density of states (spin‐DOS) with spin along the α direction is $s_{\alpha }\left(\varepsilon \right)=\left(1/\pi \right)\;\text{Im}\mathrm{Tr}\left[{\hat{\sigma }}_{\alpha }\hat{G}\left(\varepsilon ,k_{x},k_{y}\right)\right]$. δ is the typical energy broadening (≃ 10 meV) and the trace (Tr) was applied over the considered sp^3^ orbitals on a given BL index (*n* ∈ [1, *N*]). The energy zero (ε = 0) referred to the Fermi level position.

The modeling of IREE were performed by TB method. The contributions were summed from the different Fermi surface pockets within the 2D‐BZ after i) having introduced the Rashba potentials at the BiSb surfaces, required to match both the TSS electronic dispersion and SML measured in (S)ARPES experiments;^[^ ^19^ ^]^ and after ii) having considered a same (spin) relaxation time of τ_s_ = τ_0_ = 10 fs involved in the intraband transitions. The inverse Edelstein length has been then evaluated on the whole Fermi surface according to the following expression ${\Lambda^{\mathrm{IREE}}}_{\mathrm{xy}}\left(\varepsilon \right)=\frac{\sum_{n}\int d^{2}k\langle \psi_{nk}|{\hat{v}}_{x}\tau_{0}|\psi_{nk}\rangle \langle \psi_{nk}|{\hat{\sigma }}_{y}\left|\psi_{nk}\right\rangle N_{\text{DOS}}\left(\varepsilon ,k,n\right)\tau }{\sum_{n}\int d^{2}kN_{\text{DOS}}\left(\varepsilon ,k,n\right)}$ (see Section S5, Supporting Information).

The typical value of τ_s_ = 10 fs corresponded to an energy broadening Γ = ℏ/(2τ_s_) ≃ 50 meV) and a Fermi velocity of ≈5 × 10^5^ m.s^−1^ like extracted from ARPES data. The trace on κ_*xy*_ was performed by summing the BL contributions from the top surface down to the middle of the BL, typically matching the typical finite TSS evanescence or extension length.

## Conflict of Interest

The authors declare no conflict of interest.

## Author Contributions

J.‐M.G., H.J., P.LF., S.D., and A.L. conceived and designed the experiment. J.‐M.G. supervised the project. L.B., D.S., M.Mo., A.L., N.R., F.B., P.L.F., and J.‐M.G. grew the samples and performed ARPES and SARPES experiments at Synchrotron Soleil. G.P., L.B., and A.L. performed TEM cross‐section and SR‐TEM microscopy. E.R., S.D., M.Mi., J.M., and J.T. performed THz‐TDS experiments at room temperature. H.J. performed TB calculations. E.R., P.LF., S.D., A.L., R.L., N.R., H.J., and J.‐M.G. analyzed the data. E.R., P.L.F., S.D., A.L., N. R., R.L., H.J., and J.‐M.G. wrote the paper.

## Supporting information

Supporting Information

## Data Availability

The data that support the findings of this study are available from the corresponding author upon reasonable request.
