# Supplementary material for: Spin‐Momentum Locking and Ultrafast Spin‐Charge Conversion in Ultrathin Epitaxial Bi1 − x Sb x Topological Insulator
Source: Adv Sci (Weinh). 2023 Apr 25;10(19):2301124. doi: 10.1002/advs.202301124 (PMC10323647; doi:10.1002/advs.202301124)
Supplement: Supplementary file 1 — Supporting Information [file ADVS-10-2301124-s001.pdf]

ADVANCED SCIENCE Open Access

Supporting Information

for *Adv. Sci.*, DOI 10.1002/advs.202301124

Spin-Momentum Locking and Ultrafast Spin-Charge Conversion in Ultrathin Epitaxial $Bi_{1-x}Sb_x$ Topological Insulator

E. Rongione, L. Baringthon, D. She, G. Patriarche, R. Lebrun, A. Lemaître, M. Morassi, N. Reyren, M. Mičica, J. Mangeney, J. Tignon, F. Bertran, S. Dhillon, P. Le Févre, H. Jaffrès* and J.-M. George**

Supplementary Material of "Spin-momentum locking and ultrafast spin-charge conversion in ultrathin epitaxial $Bi_{1-x}Sb_x$ topological insulator."

E. Rongione,[1,2] L. Baringthon,[1,3,4] D. She,[1,3,4] G. Patriarche,[4] R. Lebrun,[1] A. Lemaître,[4] M. Morassi,[4] N. Reyren,[1] M. Mičica,[2] J. Mangeney,[2] J. Tignon,[2] F. Bertran,[3] S. Dhillon,[2,5] P. Le Fèvre,[3] H. Jaffrès,[1,5] and J.-M. George[1,5]

[1]*Unité Mixte de Physique, CNRS, Thales, Université Paris-Saclay, F-91767 Palaiseau, France*

[2]*Laboratoire de Physique de l'Ecole Normale Supérieure, ENS, Université PSL, CNRS, Sorbonne Université, Université Paris Cité, F-75005 Paris, France*

[3]*Synchrotron SOLEIL, L'Orme des Merisiers, Départementale 128, F-91190 Saint-Aubin, France*

[4]*Université Paris-Saclay, CNRS, Centre de Nanosciences et de Nanotechnologies, F-91120 Palaiseau, France*

[5]Corresponding authors: sukhdeep.dhillon@phys.ens.fr, henri.jaffres@cnrs-thales.fr, jeanmarie.george@cnrs-thales.fr

(Dated: April 13, 2023)

This Supplementary Material presents the structural properties of $Bi_{1-x}Sb_x$ derived from TEM and EDX characterization (S1), the electronic properties of $Bi_{1-x}Sb_x$ derived from ARPES (S2), the THz emission from $Bi_{1-x}Sb_x$/Co as a function of various parameters: the applied magnetic field (S3 A), the fluence dependence of the THz emission (S3 B), the THz transients renormalization procedure (S3 C), the $Bi_{1-x}Sb_x$ thickness dependence of the renormalized THz emission (S3 D), the substrate influence on the THz emission (S3 E), the THz renormalized emission as a function of the $Bi_{1-x}Sb_x$ alloy concentration (S3 F), as well as control experiments: the THz spin-injection efficiency depending on the interface quality (S4 A) or the influence of the non-magnetic capping (S4 B), and finally presents the discussions about the spin-charge conversion profile via interfacial TSS contributions and linear response theory (S5).

S1. STRUCTURAL AND ELECTRONIC PROPERTIES.

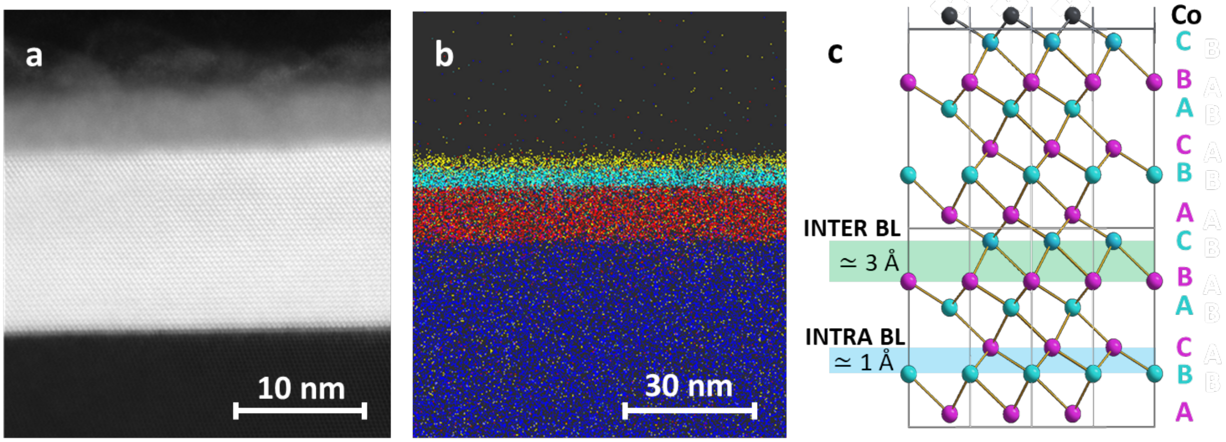

Figure FS1. **Structural properties of epitaxial $Bi_{1-x}Sb_x$ thin films on BaF_2 substrate.** (a) HAADF-STEM image of $Bi_{0.79}Sb_{0.21}$(15nm)/Co(4nm)/AlO_x(3nm) sample on BaF_2. The BaF_2 substrate is oriented following a ⟨110⟩ zone axis. (b) EDX-STEM elemental mapping of $Bi_{0.79}Sb_{0.21}$(15nm)/Co(4nm)/AlO_x(3nm) (on BaF_2 substrate) composed with Bi (red), Co (turquoise), Al (yellow) and Ba (blue) elements. (c) Graphical representation of the $Bi_{1-x}Sb_x$ bilayer decomposition, with the presence of intra-bilayer and inter-bilayer distances.

We present in this section the structural description of epitaxially grown BiSb ultrathin films. The sample studied is $Bi_{0.79}Sb_{0.21}$(15nm)/Co(2nm)/AlO_x(3nm) grown either onto BaF_2 and SiHR substrates. We report on Fig. FS1b the EDX-STEM sectional picture of the sample grown onto BaF_2 displaying the Bi, Ba, Co and Al elements. We can observe that the interfaces between *i*) the substrate and $Bi_{1-x}Sb_x$ as well as *ii*) $Bi_{1-x}Sb_x$ and Co are very smooth, demonstrating also the good epitaxial quality of the deposited BiSb layers. These are reported on Fig. FS1a for the HAADF-STEM image. For comparison, the $Bi_{1-x}Sb_x$ unit cell is graphically represented in Fig. FS1c, where each $Bi_{1-x}Sb_x$ bilayer (1 BL $\simeq$ 0.4 nm) is defined with an inter-distance about 0.3 nm and a intra-bilayer distance about 0.1 nm.

Alongside BaF_2 substrate, we have also developed the growth of $Bi_{1-x}Sb_x$ topological insulator on Si substrate which structural properties are shown in Fig. FS2. Fig. FS2 shows the EDX-STEM images obtains when growing $Bi_{0.79}Sb_{0.21}$(15nm)/Co(4nm)/AlO_x(3nm) on Si(111) substrate

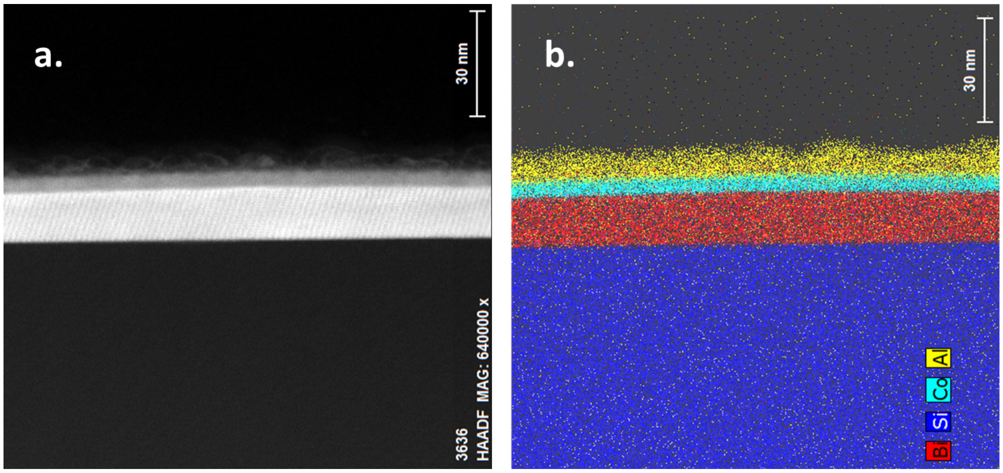

Figure FS2. **Structural properties of epitaxial $Bi_{1-x}Sb_x$ thin films on Si substrate.** (a) HAADF-STEM image of $Bi_{0.79}Sb_{0.21}$(15nm)/Co(4nm)/AlO_x(3nm) sample on Si substrate. (b) EDX-STEM elemental mapping of $Bi_{0.79}Sb_{0.21}$(15nm)/Co(4nm)/AlO_x(3nm) on Si substrate composed with Bi (red), Co (turquoise), Al (yellow) and Si (blue) elements.

and attesting of the good quality of the MBE. We observe that the interfaces composing the heterostructures, whether it is the Si/$Bi_{1-x}Sb_x$ interface or the $Bi_{1-x}Sb_x$/Co interface, are well defined and very smooth. This demonstrates the good epitaxial quality of the thin films realized on Si substrates. In particular, the quality of the Co/BiSb interface is very similar to that when the growth is on BaF_2 substrates. More details can be found in the PhD thesis of L. Baringthon [1] and in Ref. [2]. We performed complementary and quantitative EDX-STEM profile analysis (Fig. FS3) along a linescan corresponding to the layer thickness. We map a very weak interdiffusion of the chemical species and thus demonstrate very good interfaces quality.

S2. HIGH RESOLUTION SPIN-RESOLVED ARPES: SPIN TEXTURE OF Bi_xSb_{1-x} SURFACE STATES.

Fig. FS4a displays the ARPES measurements acquired on a 5 nm (12 BLs) $Bi_{0.85}Sb_{0.15}$ at room temperature. The figure displays respectively the DOS-resolved projected onto the Fermi surface (top) and the energy dispersions obtained along the $\overline{\Gamma M}$. This emphasizes, in particular, the energy band dispersions of two surface states S_1 (colored in red) and S_2 (colored in blue) as well as the *bulk* valence bands (4 bands are clearly visible).

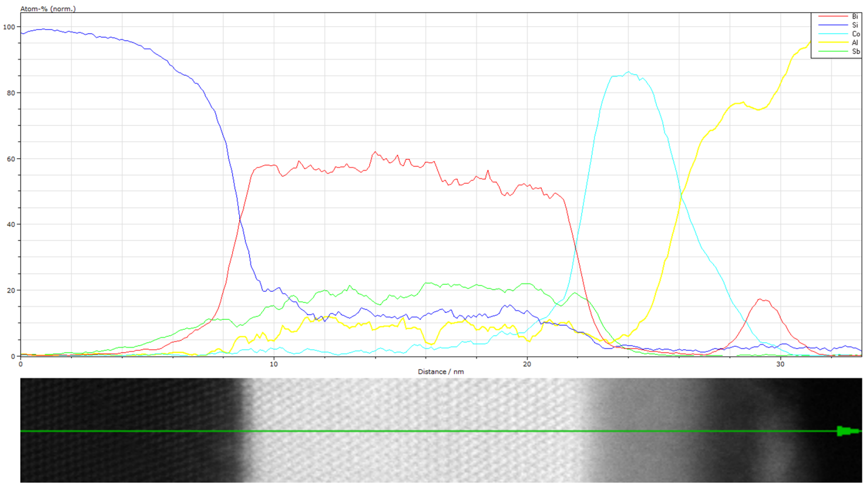

Figure FS3. **Weak chemical species interdiffusion in developed $\mathrm{Bi}_{1-x}\mathrm{Sb}_x$-based heterostructures.** Quantitative EDX-STEM linescan profile analysis of the heterostructure $\mathrm{Bi}_{0.79}\mathrm{Sb}_{0.21}$(15nm)/Co(4nm)/$\mathrm{AlO}_x$(3nm)/Carbon protective layer on Si. The chemical species tracking includes Bi (red), Si (blue), Co (turquoise), Al (yellow) and Sb (green). The bottom image presents the corresponding HAADF-STEM image.

Fig. FS4b displays the resulting 2D-DOS for the same 12 BLs $\mathrm{Bi}_{0.85}\mathrm{Sb}_{0.15}$ obtained by TB methods showing a very good agreement with ARPES data. Fig. FS4c displays the corresponding spin-resolved DOS showing large values in the same range than the DOS previously calculated. The energy splitting appears clearly in particular near the $\overline{\Gamma}$ point (DOS and spin-resolved DOS are given in arbitray units).

Fig. FS4d shows the respective values of the 2D-DOS (blue) and 2D spin-resolved DOS (red) at the Fermi level along the $\overline{\Gamma}-\overline{\mathrm{M}}$ line. The different peaks corresponds to the occurrence of the S_1 and S_2 resonances at the Fermi level (band crossed) and responsible for the SCC. One notes that the spin-DOS are asymmetric *vs.* the $\overline{\Gamma}$ point as expected emphasizing on the particular chirality of those Rashba-like states. One notes that the values of the spin-DOS represents a large fraction of the total DOS (between 50% and 100%) which corresponds to average values for $\langle\psi_{\mathbf{k}n}|\,\hat{\sigma}_y\,|\psi_{\mathbf{k}n}\rangle$ or the order of $\langle\psi_{\mathbf{k}n}|\,\hat{\sigma}_y\,|\psi_{\mathbf{k}n}\rangle \approx \pm(0.5-0.7)\,\frac{\hbar}{2}$.

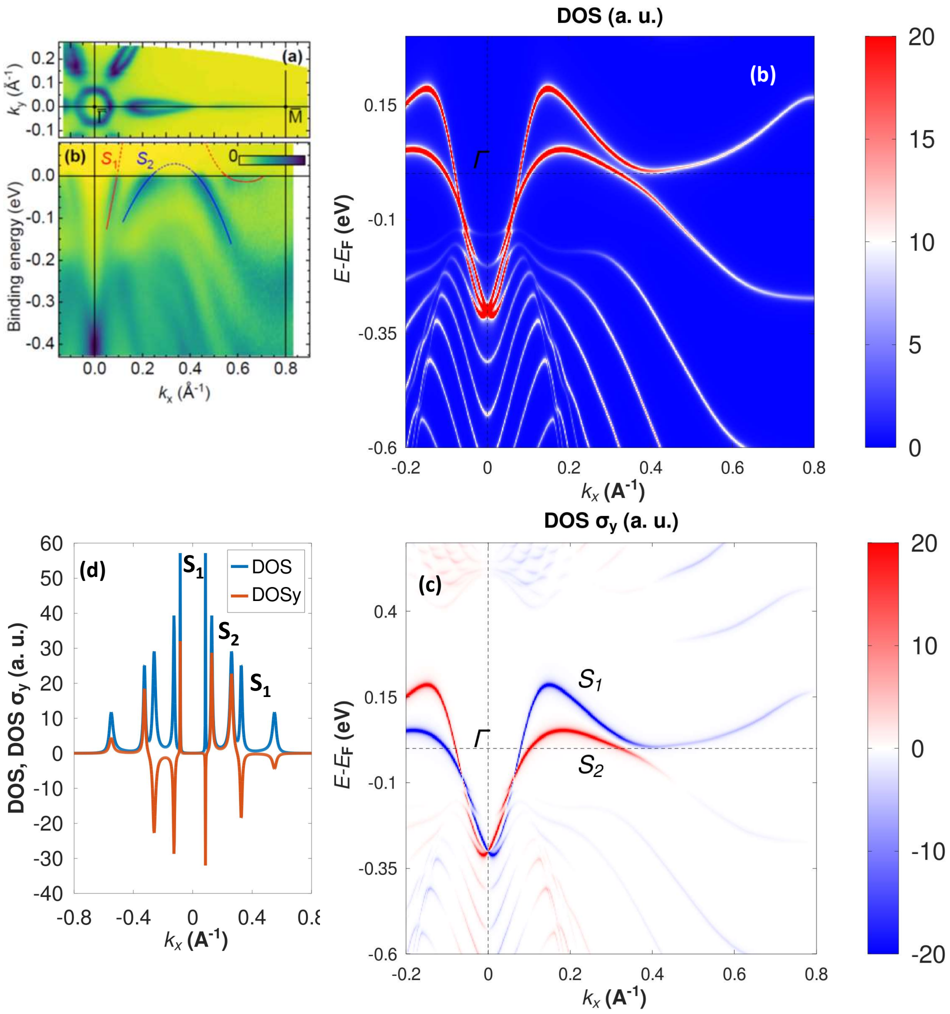

Figure FS4. **ARPES and TB results obtained from 5 nm $\mathbf{Bi_{1-x}Sb_x}$ (x=0.15).** (a) ARPES measurements along the $\overline{\Gamma \mathrm{M}}$ direction showing the energy band dispersions of two surface states S_1 and S_2 and of the *bulk* valence bands. (b) DOS TB-based calculations of the corresponding structure along the $\overline{\Gamma \mathrm{M}}$ line of the Brillouin zone and projected onto the first BL. (c) Corresponding spin-resolved DOS calculated by the TB method and projected onto the first BL. (d) Plot of both DOS and spin-resolved DOS projected onto the first BL showing the large spin-polarization along $\overline{\Gamma \mathrm{M}}$.

S3. THz EMISSION FROM $Bi_{1-x}Sb_x$/Co.

A. THz emission *vs.* the magnetic field and dipolar emission.

We present on Fig. FS5 the THz emission obtained from $Bi_{0.85}Sb_{0.15}(5)/Co(2)$ as a function of the magnetic field angle $\mathbf{B}(\theta)$ in polar plot. The blue region indicates a positive polarity (or phase) of the THz timetrace whereas the red color indicates a negative phase in agreement with the convention of Fig. 2 of the main text. We recover then the expected dipolar emission (with a characteristic sinus shape) owing to the cross product $\mathcal{J}_c \propto (\mathcal{J}_s, \mu_s) \times \mathbf{M}$ between the spin current j_s or spin accumulation (spin density) μ_s and the in-plane magnetization $\mathbf{M}(\theta)$ (convoluted with the detection direction of the electro-optic detector). The presence of symmetrical emission lobes indicates an absence of additional contributions to the THz emission (*i.e.* non-magnetic contributions).

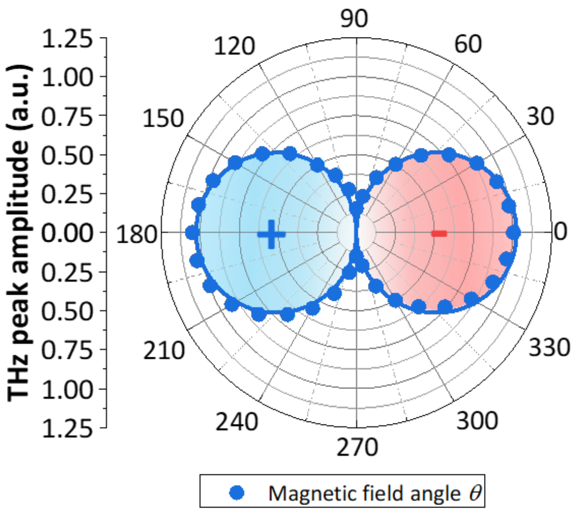

Figure FS5. **THz emission from $Bi_{1-x}Sb_x(5, x=0.15)/Co(2)/AlO_x(3)$ as a function of the magnetic field angle $\mathbf{B}(\theta)$.** The magnetic field is applied in the sample plane with an angle θ from the y-axis. The measurements have been performed at room temperature (RT).

B. Fluence dependence on the THz emission.

We report on Fig. FS6 the fluence dependence of $Bi_{0.93}Sb_{0.07}(5nm)/Co(2nm)/AlO_x(3nm)$ for laser pump power ranging from 0 to 350 mW. We observe a linear dependence of the type $E_{THz} \propto$

P_{NIR} in agreement with the absorption/excitation process and subsequent spin-charge conversion [3]. No saturation of the THz amplitude with the pump fluence could be observe, we thus place our study below both damage and saturation thresholds.

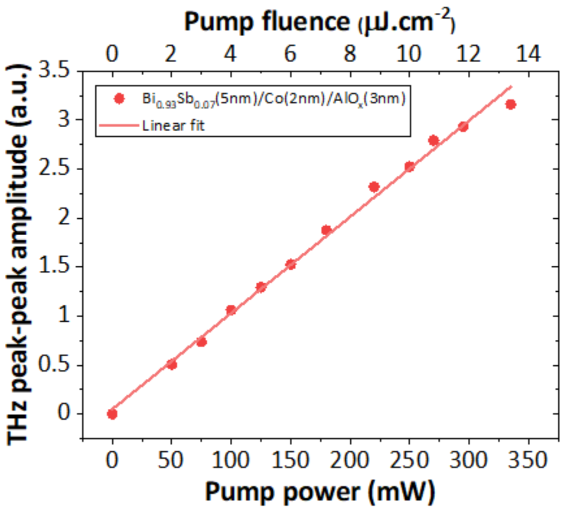

Figure FS6. **Fluence dependence on the THz emission.** THz peak-peak amplitude from $Bi_{0.93}Sb_{0.07}$(5nm)/Co(2nm)/AlO_x(3nm) on SiHR as a function of the pump power and fluence. The measurements have been performed at RT.

C. THz renormalization procedure by withdrawing the optical absorptions.

We followed the method proposed in Refs. [4–6]. We extract, from the measured THz electric field $\mathbf{E}_{\mathrm{THz}}$, the THz efficiency η_{THz} directly linked to the spin-charge conversion and spin-injection properties according to:

$$|\mathbf{E}_{\mathrm{THz}}| \propto \left(\frac{Z_0 \times \mathcal{A}_{\mathrm{NIR}}}{1 + n_{\mathrm{sub}}(\omega) + Z_0 \sum_i \sigma_i(\omega) t_{\mathrm{i}}} \right) \times (\alpha_{\mathrm{SCC}}\ \eta_{\mathrm{THz}}) \qquad \text{(ES1)}$$

where $Z_0 = 377\ \Omega$ is the free-space impedance, $\sigma_i = 1/\rho_i$ is the layer conductivity (obtained from the resistivity ρ_i), t_i is the layer thickness, α_{SCC} is the spin-charge conversion efficiency and n_{sub} is the THz refractive index of the substrate (presently $n_{\mathrm{sub}}^{\mathrm{BaF_2}} = 2.7$ [7] and $n_{\mathrm{sub}}^{\mathrm{Si}} = 3.2$, considered constant over the studied frequency range from 0.3 to 3 THz). Concerning the conductivity, we considered a Drude model approach for the AC conductivity of the thin metallic layers, such as $\sigma(\omega) =$

$\sigma_0/(1+i\omega\tau)$ where σ_0 is the DC conductivity, ω is the frequency and τ is the (collision) relaxation time. In our case, the THz frequencies are below the Drude (plasmon) frequencies of the considered materials, which allows us to consider the DC conductivity instead. The near-infrared (NIR) radiation absorption in thin films is modelled via Beer-Lambert absorption $\mathcal{A}_{\mathrm{NIR}} = \prod_i \exp(-\alpha_i t_i)$ where α_i is the absorption coefficient. The considered values for the studied heterostructures are collected in Table TS1.

**Material**	**Resistivity ρ (µΩ.cm)**	**Absorption coefficient α (cm^{-1})**
Co	29	7.5×10^5 [8]
Pt	30	7.7×10^5 [8]
Au	25	9.7×10^5 [9]
W	25	4.3×10^5 [8]
Al	40	1.3×10^6 [8]
AlO_x	-	1.15×10^6 [10]
$Bi_{1-x}Sb_x$	560	-

Table TS1. Considered values for the resitivities ρ and the absorption coefficients α (at pump wavelength λ_{NIR}=800 nm) depending on the material. Resistivity values are obtained from magneto-transport measurements.

D. Renormalization of the THz emission as a function of the $Bi_{1-x}Sb_x$ thickness.

In this section, we present the THz emission and renormalized THz efficiency η_{THz} for two thickness-dependent series, either *i)* $t_{\mathrm{TI}} = \{2.5, 5, 15\}$ nm for $x = 0.21$ (presented in the main text in Fig. 2c) and *ii)* $t_{\mathrm{TI}} = \{5, 50\}$ nm for $x = 0.15$. In the first case whose results are displayed in Fig. FS7, we present the THz emission and THz efficiency η_{THz} for $Bi_{1-x}Sb_x(t_{\mathrm{TI}})$/Co(4nm)/Au(4-6nm) grown on SiHR substrate. We observe an almost independent THz efficiency as a function of the $Bi_{1-x}Sb_x$ thickness, in favor of an interfacially-mediated spin-charge conversion process. The efficiency is similar to the Co(2)/Pt(4) reference.

Secondly, we present on Fig. FS8 the THz signal from $Bi_{0.85}Sb_{0.15}(t_{\mathrm{TI}})$/Co(2)/$AlO_x$(3nm) (second Sb content series grown on SiHR substrate) with t_{TI} ranging from 5 to 50 nm. The THz emission from 5 nm-thick sample reach up to ×1.5 the emission from reference Co(2)/ Pt(4). Both

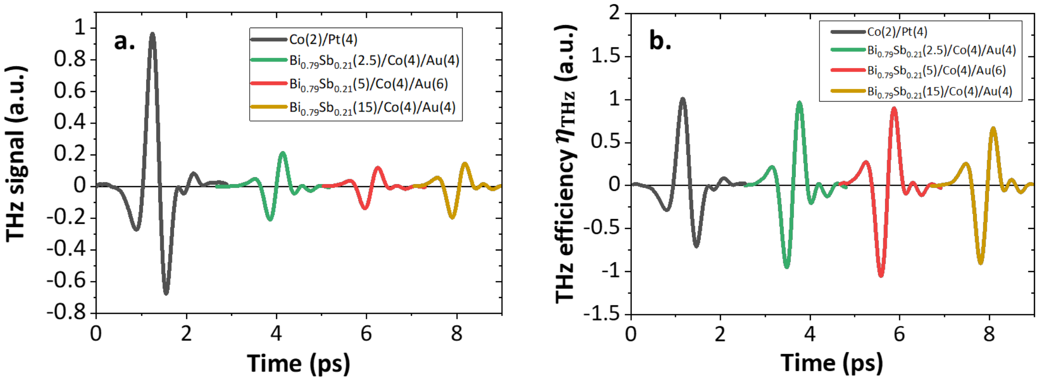

Figure FS7. **Renormalization procedure of the THz emission for the thickness-dependent series.** (a) THz emission and (b) THz efficiency of $Bi_{1-x}Sb_x(t_{TI})$/Co(4nm)/Au(4-6nm) grown on SiHR substrate as a function of $Bi_{1-x}Sb_x$ thickness from $t_{TI} = \{2.5, 5, 15\}$ nm (and Sb content $x = 0.21$). *Time traces are shifted in time for clarity.*

signal and THz efficiency η_{THz} of the 50 nm-thick sample is reduced compared to the 5 nm-thick $Bi_{1-x}Sb_x$ sample, respectively by a factor 2.2 and 1.9.

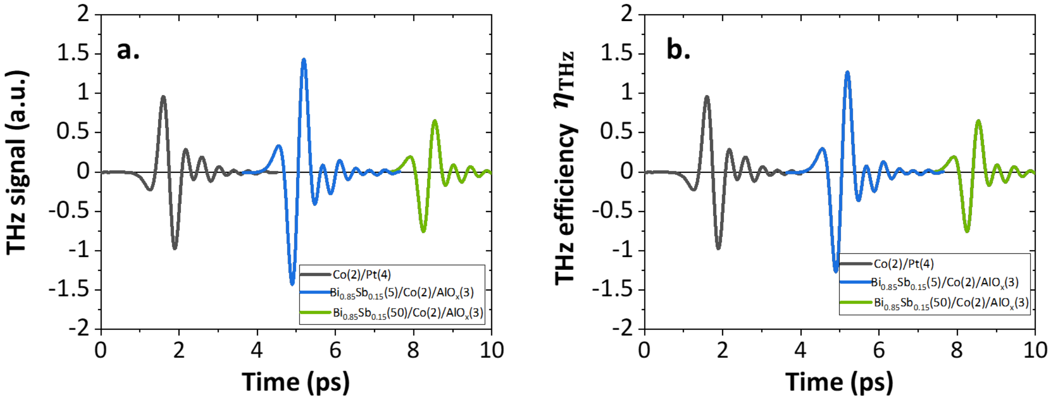

Figure FS8. **$Bi_{1-x}Sb_x$ thickness dependence on the THz emission.** (a) Measured THz signals and (b) renormalized THz efficiency η_{THz} from $Bi_{0.85}Sb_{0.15}$(5nm)/Co(2nm)/AlO_x(3nm) and $Bi_{0.85}Sb_{0.15}$(50nm)/Co(2nm)/AlO_x(3nm) compared to Co(2nm)/Pt(4nm). The substrate is SiHR. *Time traces are shifted in time for clarity.*

E. Substrate dependence on the THz emission from $Bi_{1-x}Sb_x$/Co bilayers.

Fig. FS9 displays the impact of the substrate on both THz emission and THz efficiency η_{THz} for identical $Bi_{0.79}Sb_{0.21}$(15nm)/Co(4nm)/AlO_x(3nm) (or Au(4nm)) structures. The THz signal is larger on BaF_2 substrates; however a larger THz efficiency η_{THz} is observed on SiHR substrates on which we mainly focus in the present work. This is explained by the difference in THz refractive index between the substrates [7]. Thus, in the remaining part of the study, we exclude the use of BaF_2 substrate as it can lead to *i*) non-linear optical contributions to the THz emission and to *ii*) charging effects in (S)ARPES experiments.

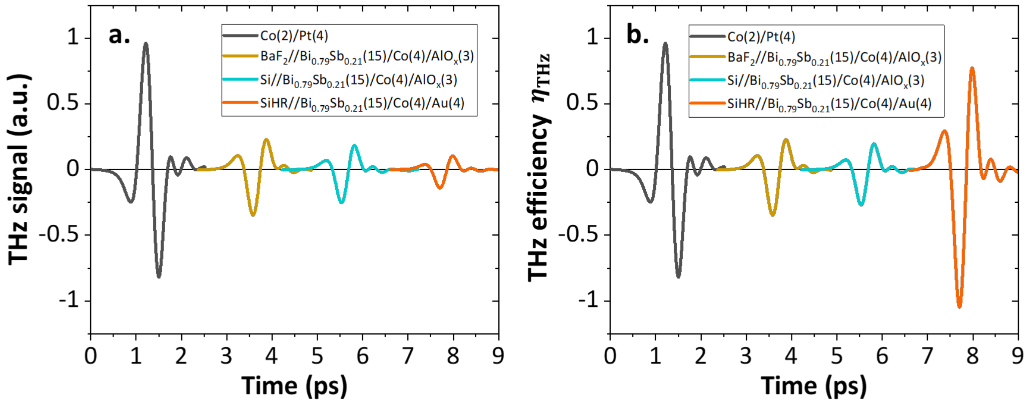

Figure FS9. **Impact of the substrate on the THz emission.** (a) THz emission and (b) THz efficiency η_{THz} of $Bi_{0.79}Sb_{0.21}$(15nm)/Co(3nm)/AlO_x(3nm) (or Au(4nm)) as a function of the substrate (either BaF_2, Si or SiHR) compared to Co(2nm)/Pt(4nm). *Time traces are shifted in time for clarity.*

F. Renormalization of the THz emission as a function of the alloy ratio $Bi_{1-x}Sb_x$.

In this section, we present the raw THz emission (prior to any renormalization treatment) and efficiency η_{THz} (after renormalization) from $Bi_{1-x}Sb_x$/Co alloy as a function of the Sb concentration for the two sample series: *i*) $Bi_{1-x}Sb_x$(15)/Co(4) for Sb concentration $x = \{0.1, 0.21, 0.3, 0.4\}$ (in Fig. FS10) and *ii*) $Bi_{1-x}Sb_x$(5)/Co(2) for Sb concentration $x = \{0.07, 0.15, 0.3\}$ (in Fig. FS11). Surprisingly, we observe that a sizeable THz contribution is recovered at $x = 0.4$, typically out

of the topological window [11, 12], thus possibly addressing the role of spin-textured Rashba-like hybridized surface states to the overall THz spin-charge conversion.

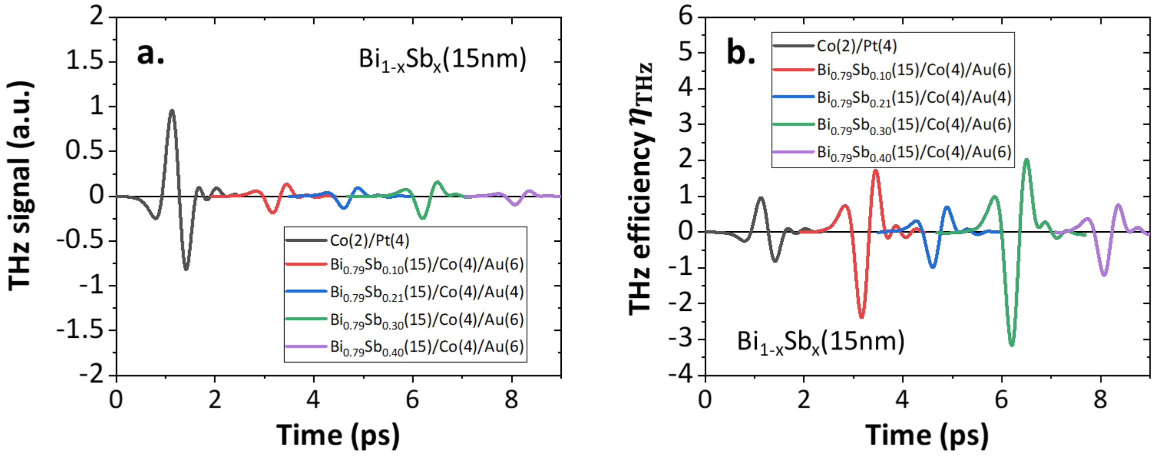

Figure FS10. **$Bi_{1-x}Sb_x$ alloy dependence.** (a) THz emission and (b) THz efficiency of $Bi_{1-x}Sb_x(15)/Co(4)/Au$ as a function of the Sb concentration, presently $x = \{0.1, 0.21, 0.3, 0.4\}$ compared to Co(2)/Pt(4). *Time traces are shifted in time for clarity.*

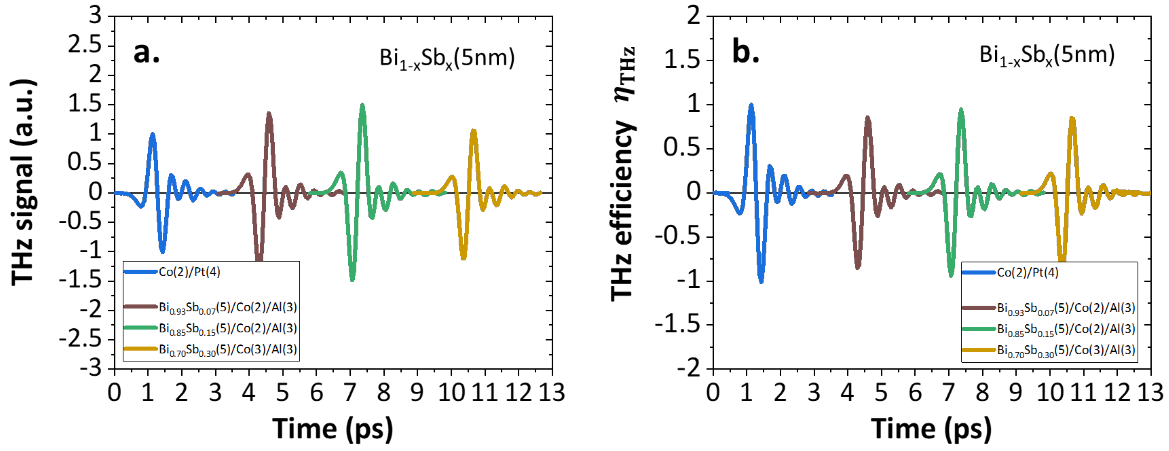

Figure FS11. **$Bi_{1-x}Sb_x$ alloy dependence.** (a) Measured THz emission and (b) THz efficiency of $Bi_{1-x}Sb_x(5)/Co(2)/AlO_x(3)$ as a function of the Sb concentration, presently $x = \{0.07, 0.15, 0.30\}$ compared to Co(2)/Pt(4). *Time traces are shifted in time for clarity.*

G. Temperature dependence on the THz emission from $Bi_{1-x}Sb_x$/Co bilayers.

We investigated the temperature dependence of the THz emission from $Bi_{0.79}Sb_{0.21}$(15nm)/Co(4nm)/Au(4nm) on SiHR from 4 K to 300 K as shown in Fig. FS12. We can see that at low temperature, the THz signal decreases up to -14% compared to room temperature. This can be attributed to *i*) the change of the conductivity of the metallic capping (Pt and Au) with temperature [13] or a decrease of the $Bi_{1-x}Sb_x$ resistivity with temperature [2]. Both situations would lead to an increase of the THz absorption scaling with $1/Z(\omega)$ and thus a decrease of the THz signal. However, as the resistivity of $Bi_{1-x}Sb_x$ layer is about an order of magnitude higher than the Pt and Au metallic cappings, we assume that the main contribution to the change of resistivity comes from the top metallic cappings.

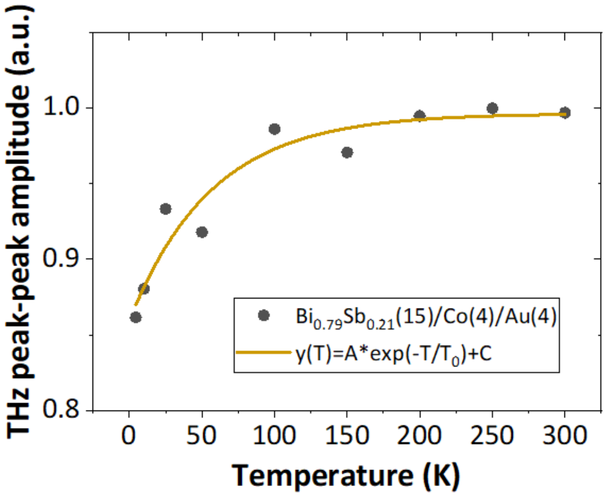

Figure FS12. **Temperature dependence on the THz emission from $Bi_{1-x}Sb_x$/Co.** THz peak-peak amplitude from $Bi_{0.79}Sb_{0.21}$(15nm)/Co(4nm)/Au(4nm) on SiHR substrate as a function of the temperature from 4 K to 300 K.

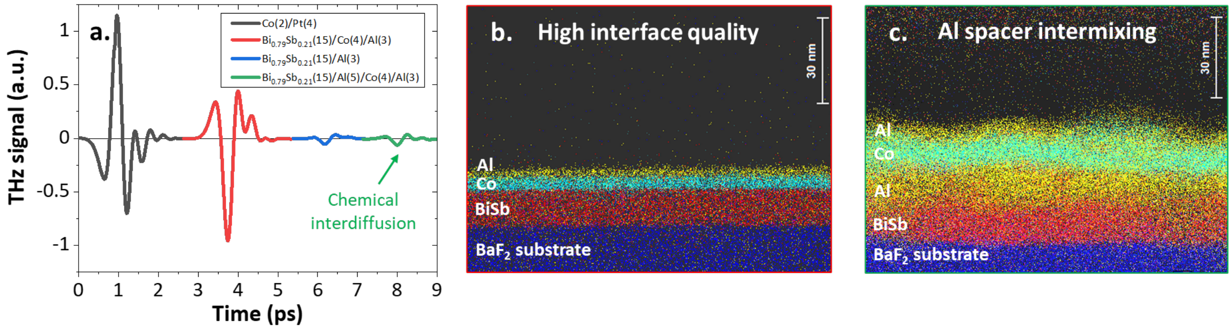

Figure FS13. **Interfacially-mediated THz emission from $Bi_{1-x}Sb_x$/Co.** (a) THz emission collected on $Bi_{1-x}Sb_x$/Co bilayer, $Bi_{1-x}Sb_x$/AlO_x and $Bi_{1-x}Sb_x$/Al/Co/Al compared to Co/Pt reference. (b-c) Transmission electron microscopy (TEM) images in case of (b) $Bi_{1-x}Sb_x$/Co/Al and (c) $Bi_{1-x}Sb_x$/Al/Co/Al samples. The interface is strongly degraded in the latter due to Al spacer intermixing, leading to possible interfacial SCC destruction. *Time traces are shifted in time for clarity.*

S4. ADDITIONAL THz EXPERIMENTS ON CONTROLLED SAMPLES: ABSENCE OF SELF-EMISSION, EFFECT OF INTERFACE DEGRADATION AND CAPPING.

A. Interface degradation and THz emission.

In order to investigate the origin of the spin-charge conversion and THz generation, we performed complementary measurements on two additional samples: one sample free of any ferromagnetic Co (only capped with naturally oxidized AlO_x(3)) and one sample with a metallic Al(5) spacer adding between the TI $Bi_{1-x}Sb_x$(15) and the ferromagnetic Co(4). We shown in Fig. FS13 their THz emission compared to the metallic Co/Pt reference and the emission from $Bi_{1-x}Sb_x$/Co. Clearly, owing to the THz signal level from the bare BiSb sample free of Co, the THz emission in $Bi_{1-x}Sb_x$/Co mainly originates from SCC at Co/BiSb interfaces. Moreover, once a thin metallic Al spacer is inserted between $Bi_{1-x}Sb_x$ and Co, the resulting emission is strongly reduced at the same level of the bare $Bi_{1-x}Sb_x$ sample. Several hypothesis would explain this emission reduction: *i*) NIR and THz absorptions in the Al spacer, *ii*) degradation of the spin-injection efficiency and/or *iii*) a reduction of the interfacial spin-charge conversion efficiency. In more details, from Table TS1, we can

estimate the differential absorption from an additional Al(5) layer that we evaluate at $\mathcal{A}'_{\mathrm{NIR}} \simeq 0.52$ and $\mathcal{Z}'_{\mathrm{THz}} \simeq 0.7$ respectively for the NIR and THz radiations. It leads to a down-scaling of the THz emission from $\mathrm{Bi}_{1-x}\mathrm{Sb}_x$/Al/Co/Al of about $\times 3$ while experimentally a reduction by a factor $\times 14$ is observed. Then a major part of the signal difference (factor $\times 5$) originates either from a loss of the spin-injection and/or a loss of spin-charge conversion efficiencies.

B. Impact of the capping on the THz emission from $\mathbf{Bi}_{1-x}\mathbf{Sb}_x$/Co bilayers.

To exclude the contributions from the capping layers (AlO_x, Au and Co), we performed reference measurements on Co(2)/AlO_x(3) and Co(4)/Au(4) as presented in Fig. FS14. We observe that both signals are small in amplitude compared to the Co/Pt reference, about an order of magnitude smaller. The small THz contribution could arise from the anomalous Hall effect in Co.

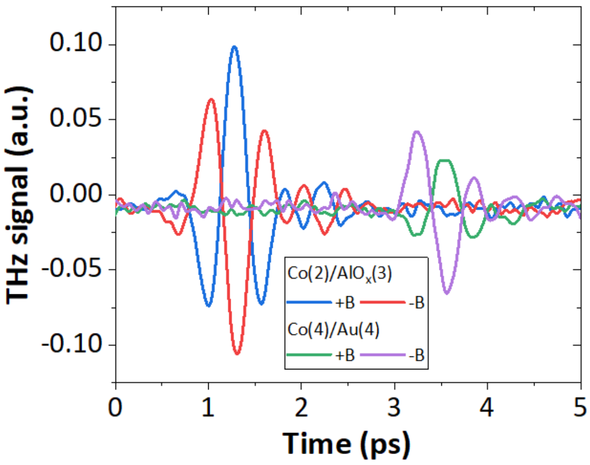

Figure FS14. **Capping reference THz measurements.** THz emission from Co(2)/AlO_x(3) and Co(4)/Au(4) normalized with respect to Co(2)/Pt(4). *Time traces are shifted in time for clarity.*

S5. LINEAR RESPONSE THEORY FOR RASHBA-EDELSTEIN EFFECT.

In this section, we develop the linear response theory to describe the Inverse Rashba-Edelstein response from the generalized Kubo formula applied to BiSb ultrathin films. We treat first *i*) the tight-binding modelling of BiSb films before tackling *ii*) the calculations of the inverse Rashba-Edelstein tensor obtained from the linear response theory, before evidencing *iii*) the (crystallographic) angular response of the spin-charge conversion to address the IREE contribution via the surface states of $Bi_{1-x}Sb_x$, as discussed previously in Ref. [14] and in agreement with (S)ARPES measurements.

A. Linear response theory in electronic transport.

We present here the linear response theory and principle of the Rashba-Edelstein response. Generally, one searches for the response function of a given operator $\hat{\mathcal{O}}$ under a certain excitation described by an interaction Hamiltonian $\hat{\mathcal{H}}_{int} = \int \hat{\mathcal{B}} F(t) d^3r dt$ where $\hat{\mathcal{B}}$ is a quantum operator and $F(t)$ is a classical source. One can then divide the response function $\mathcal{R}$ with $< \hat{\mathcal{O}} > (\omega) = \mathcal{R} F(\omega)$ (ω is the frequency) *via* respective *intraband* and *interband* terms from the respective Fermi sea and Fermi surface contributions. One obtain for $\omega = 0$ in the limit of low frequency (Drude model):

$$\mathcal{R}^{I(a)} = \frac{e^2}{2\pi V} \operatorname{Tr} < \hat{\mathcal{O}} G^R(\epsilon_{\mathrm{F}}) \hat{\mathcal{B}} G^A(\varepsilon_{\mathrm{F}}) > \quad \text{(ES2)}$$

$$\mathcal{R}^{I(b)} = -\frac{e^2}{4\pi V} \operatorname{Tr} < \hat{\mathcal{O}} G^R(\varepsilon_{\mathrm{F}}) \hat{\mathcal{B}} G^R(\varepsilon_{\mathrm{F}}) > +c.c. \quad \text{(ES3)}$$

$$\mathcal{R}^{II} = \frac{e^2}{4\pi V} \int_{-\infty}^{+\infty} f(\varepsilon) d\varepsilon \operatorname{Tr} < \left[\hat{\mathcal{O}} G^R \hat{\mathcal{B}} \frac{\partial G^R}{\partial \varepsilon} - \hat{\mathcal{O}} \frac{\partial G^R}{\partial \varepsilon} \hat{\mathcal{B}} G^R \right] > +c.c. \quad \text{(ES4)}$$

where $G^{R,A} = \frac{1}{\varepsilon - \hat{\mathcal{H}} \pm i\Gamma}$ are the respective retarded (R) and advanced (A) Green's functions. The intraband response is given by the sum of the two terms, $\mathcal{R}^{I(a)} + \mathcal{R}^{I(b)}$ with $\mathcal{R}^{I(a)}$ generally considered as much larger for clean systems whereas the interband term (Fermi sea) is given by $\mathcal{R}^{II}$.

1. *Intrinsic Inverse spin Hall effect from the linear response theory. [15, 16]*

For ISHE, we are searching for the expression of the 2D in-plane current density $\mathcal{J}_c$ along the x direction when a spin-current is injected. The interaction term is $\hat{\mathcal{H}}_{int} = \int \hat{\mathcal{J}}_s E(t) d^3r dt$ where E is

the electric field and $\hat{\mathcal{J}}_s$ is the spin current. Starting from the Kubo formula, we obtain:

$$\sigma_{xy}^{I(a)} = \frac{e^2}{2\pi V} \mathrm{Tr} < \hat{v}_y G^R(\varepsilon_\mathrm{F}) \hat{v}_x G^A(\varepsilon_\mathrm{F}) > \tag{ES5}$$

$$\sigma_{xy}^{I(b)} = -\frac{e^2}{4\pi V} \mathrm{Tr} < \hat{v}_y G^R(\varepsilon_\mathrm{F}) \hat{v}_x G^R(\varepsilon_\mathrm{F}) > +c.c. \tag{ES6}$$

$$\sigma_{xy}^{II} = \frac{e^2}{4\pi V} \int_{-\infty}^{+\infty} f(\varepsilon) d\varepsilon \, \mathrm{Tr} < \left[\hat{v}_y G^R \hat{v}_x \frac{\partial G^R}{\partial \varepsilon} - \hat{v}_y \frac{\partial G^R}{\partial \varepsilon} \hat{v}_x G^R \right] > +c.c. \tag{ES7}$$

We write the intrinsic contribution to the spin-Hall conductivity after having introduced the velocity operator $\hat{\mathbf{v}} = \partial \hat{\mathcal{H}} / \partial \mathbf{p}$, and the spin current operator $\hat{\mathcal{J}}_{zy} = \{ \hat{\mathbf{v}}_z \hat{\sigma}_y \}$ according to:

$$\sigma_{xz}^y = \frac{e^2 \hbar}{2\pi} \sum_{n \; ; \; m \neq n} \int_{\mathrm{BZ}} (f_n(\mathbf{k}) - f_m(\mathbf{k})) \frac{[\langle \psi_{n\mathbf{k}} | \, \hat{v}_x \, | \psi_{m\mathbf{k}} \rangle \, \langle \psi_{m\mathbf{k}} | \, \{ \hat{\sigma}_y \hat{v}_z \} \, | \psi_{n\mathbf{k}} \rangle]}{(\varepsilon_{n\mathbf{k}} - \varepsilon_{m\mathbf{k}})^2 + \Gamma^2} \frac{d^2\mathbf{k}}{(2\pi)^2} \tag{ES8}$$

where e the electron charge, $\hbar$ the reduced Planck constant, $\Gamma = \hbar / 2\tau$ the typical energy broadening, $|n\rangle$ and $|m\rangle$ the quantum states for the wavevector $\mathbf{k}$. $\psi_{i\mathbf{k}}$ is the electronic state associated with energy $\varepsilon_{i\mathbf{k}}$ and $f_i(\mathbf{k})$ is the Fermi filling factor.

Note however that the ISHE gives zero contribution to the charge current for non-propagating states along the direction normal to the layers that is the case in the bandgap of the BiSb layers.

2. Inverse Rashba-Edelstein tensor from the linear response theory.

We have adapted this formalism to the following interaction term $\hat{\mathcal{H}}_{int} = -\int \hat{\mathcal{M}} \dot{B}(t) d^2r dt$ where $\hat{\mathcal{M}} = \frac{e\hbar}{m^*} \hat{\sigma}$ and B is the magnetic field according to the formalism developed by Shen *et al.* [17]. We have then expanded this framework to the inverse Rashba-Edelstein tensor $\Lambda_{xy}^{\mathrm{IEE}} = \mathcal{J}_c / \mathcal{J}_s$ where $\mathcal{J}_c$ is the 2D in-plane charge current along x whereas $\mathcal{J}_s = \mathcal{J}_{sz}^y$ is the 3D ongoing spin-current with flux directed along the direction z normal to the layer carrying a spin σ_y. Starting from the Kubo formula and retaining the $I(a)$ term only (intraband or Fermi surface term) for the intrinsic spin-Hall conductivity described here for clean epitaxial samples:

We find respectively the local charge-current $\mathcal{J}_{c\mathbf{k}}$ and local magnetization density $\mu_{\mathbf{k}}^y$ generated by $\dot{B}_y$:

$$\mathcal{J}_{ck}(\varepsilon) = \frac{e^2\hbar^2}{2\pi m^*\Gamma}\sum_n f_{\mathbf{k}n}(\varepsilon)\frac{[\langle\psi_{n\mathbf{k}}|\,\hat{v}_x\,|\psi_{n\mathbf{k}}\rangle\,\Gamma\,\langle\psi_{n\mathbf{k}}|\,\hat{\sigma}_y\,|\psi_{n\mathbf{k}}\rangle]}{(\varepsilon-\varepsilon_{n\mathbf{k}})^2+\Gamma^2}\dot{B}$$
$$= \frac{e^2\hbar}{m^*}\sum_n f_{\mathbf{k}n}(\varepsilon)\,\langle\psi_{n\mathbf{k}}|\,\hat{v}_x\tau_{\mathbf{k}n}\,|\psi_{n\mathbf{k}}\rangle\,\langle\psi_{n\mathbf{k}}|\,\hat{\sigma}_y\,|\psi_{n\mathbf{k}}\rangle\,\mathcal{N}_{\mathrm{DOS}}(k,\varepsilon)\dot{B} \quad \text{(ES9)}$$
$$\mu_k(\varepsilon) = \frac{e\hbar^2}{2\pi m^*\Gamma}\sum_n f_{\mathbf{k}n}(\varepsilon)\frac{[\langle\psi_{n\mathbf{k}}|\,\hat{\sigma}_y\,|\psi_{n\mathbf{k}}\rangle\,\Gamma\,\langle\psi_{n\mathbf{k}}|\,\hat{\sigma}_y\,|\psi_{n\mathbf{k}}\rangle]}{(\varepsilon-\varepsilon_{n\mathbf{k}})^2+\Gamma^2}\dot{B}$$
$$= \frac{e\hbar}{m^*}\sum_n f_{\mathbf{k}n}(\varepsilon)\,\langle\psi_{n\mathbf{k}}|\,\hat{\sigma}_y\,|\psi_{n\mathbf{k}}\rangle\,\langle\psi_{n\mathbf{k}}|\,\hat{\sigma}_y\,|\psi_{n\mathbf{k}}\rangle\,\mathcal{N}_{\mathrm{DOS}}(k,\varepsilon)\tau_{\mathbf{k}n}\dot{B} \quad \text{(ES10)}$$

where $\tau_{\mathbf{k}n} = \tau_0\,\langle\psi_{n\mathbf{k}}|\,\hat{\sigma}_y\,|\psi_{n\mathbf{k}}\rangle^2$ is the characteristic spin-relaxation time at point $\mathbf{k}$ for spin oriented along the y direction injected onto the subband n in the Brillouin zone. This specific time $\tau_{\mathbf{k}}$ ($\mathbf{k}$-dependent) scales with characteristic time τ_0 (the mobility time) and takes into account the possible fast-relaxation at point $\mathbf{k}$ when the spin-direction (y) does not match with the corresponding eigenvector spin direction of the band. Spin-current $\mathcal{J}^y_{s\mathbf{k}}$ and $\mu^y_{\mathbf{k}}$ are linked by $\mu^y_{\mathbf{k}} = \mathcal{J}^y_{s\mathbf{k}}\tau_{\mathbf{k}}$ whereas $\mathcal{J}^y_{s\mathbf{k}} = \frac{\sum_n \mathcal{N}_{\mathrm{DOS}}(\varepsilon,\mathbf{k},n)}{\sum_n\int\mathcal{N}_{\mathrm{DOS}}(\varepsilon,\mathbf{k},n)d^2k}\mathcal{J}_s$. We thus obtain:

$$\mathcal{J}^x_c(\varepsilon) = \frac{\sum_n\int d^2\mathbf{k}\,\langle\psi_{n\mathbf{k}}|\,\hat{v}_x\tau_0\,|\psi_{n\mathbf{k}}\rangle\,\langle\psi_{n\mathbf{k}}|\,\hat{\sigma}_y\,|\psi_{n\mathbf{k}}\rangle\,\mathcal{N}_{\mathrm{DOS}}(\varepsilon,\mathbf{k},n)}{\sum_n\int d^2\mathbf{k}\mathcal{N}_{\mathrm{DOS}}(\varepsilon,\mathbf{k},n)}\mathcal{J}^y_{sz} \quad \text{(ES11)}$$

which defines thus the IREE tensor Λ_{xy} at the same time that the average IREE length.

$$\Lambda^{\mathrm{IEE}}_{xy}(\varepsilon) = \frac{\sum_n\int d^2\mathbf{k}\,\langle\psi_{n\mathbf{k}}|\,\hat{v}_x\tau_0\,|\psi_{n\mathbf{k}}\rangle\,\langle\psi_{n\mathbf{k}}|\,\hat{\sigma}_y\,|\psi_{n\mathbf{k}}\rangle\,\mathcal{N}_{\mathrm{DOS}}(\varepsilon,\mathbf{k},n)}{\sum_n\int d^2\mathbf{k}\mathcal{N}_{\mathrm{DOS}}(\varepsilon,\mathbf{k},n)} \quad \text{(ES12)}$$

We note that for the case of topological states represented by a single dispersion cone corresponding to the band n, Λ_{xy} reduces to $\Lambda_{xy} = \langle\psi_{n\mathbf{k}}|\,\hat{v}_x\tau_0\,|\psi_{n\mathbf{k}}\rangle\,\langle\psi_{n\mathbf{k}}|\,\hat{\sigma}_y\,|\psi_{n\mathbf{k}}\rangle$ as expected.

Projecting the in-plane wavevector $\mathbf{k} := (k\cos(\phi-\phi_{\mathrm{R}}), k\sin(\phi-\phi_{\mathrm{R}}))$ onto a particular crystallographic direction (*e.g.* $\phi_{\mathrm{R}} = 0 \Leftrightarrow \overline{\Gamma\mathrm{M}}$ in the reciprocal space), one finally obtains:

$$\Lambda^{\mathrm{IEE}}_{xy}(\theta,\phi_{\mathrm{R}}) = \frac{\hbar^3}{2\pi m^*}\int_{\mathrm{BZ}} d\phi\;\hat{v}_x(\phi,\phi_{\mathrm{R}})\hat{\sigma}_y(\phi,\theta,\phi_{\mathrm{R}})\sum_n\frac{f_n(k)d^2k}{\Gamma^2}$$
$$= \frac{\hbar^3}{2\pi m^*\Gamma}\int_0^{2\pi} d\phi\;\hat{v}_x(\phi,\phi_{\mathrm{R}})\hat{\sigma}_y(\phi,\theta,\phi_{\mathrm{R}})\left(\frac{d^2k}{d\varepsilon}\right) \quad \text{(ES13)}$$
$$= \frac{\hbar^3}{2\pi m^*\Gamma}\int_0^{2\pi} d\phi\;\hat{v}_x(\phi,\phi_{\mathrm{R}})\hat{\sigma}_y(\phi,\theta,\phi_{\mathrm{R}})\mathcal{N}_{\mathrm{DOS}}(\phi,\phi_{\mathrm{R}})$$

where we have introduced the density of states $\mathcal{N}_{\mathrm{DOS}}(\varepsilon) = (d^2k/d\varepsilon)$, θ the orientation of out-of-equilibrium injected spins (in-plane magnetization or magnetic field), ϕ_{R} the in-plane sample

orientation and ϕ the generic azimuthal angle. Therefore, the conversion symmetries are given by the integrated product between the longitudinal group velocity, the spin orientation, and the density of states. For C_{3v} symmetry systems, the energy dispersion relation including Rashba term [18–20] reads:

$$\varepsilon_{\pm}(\mathbf{k}) = \frac{\hbar^2 k^2}{2m} \pm \sqrt{\alpha_{\mathrm{R}}^2 k^2 + \lambda^2 k^6 \cos^2(3\phi)} \tag{ES14}$$

with α_{R} the Rashba coefficient (velocity) and λ a coefficient describing the strength of the six-fold anisotropy term. Thus we assume the spin accumulation, group velocity and density of states to have the following symmetries:

$$\begin{aligned} v_x(\phi, \phi_{\mathrm{R}}) &\equiv \cos(\phi) + 3\lambda \cos(3(\phi - \phi_{\mathrm{R}})) \left[\cos(\phi_{\mathrm{R}}) \cos(\phi - \phi_{\mathrm{R}}) + \sin(\phi_{\mathrm{R}}) \sin(\phi - \phi_{\mathrm{R}})\right] \\ \sigma_y(\phi, \theta, \phi_{\mathrm{R}}) &\equiv \frac{1}{2}\left(1 + \sin(\phi + \phi_{\mathrm{M}})\right) \times \left(1 + 3\beta \cos^2(3(\phi - \phi_{\mathrm{R}}))\right) \\ \mathcal{N}_{\mathrm{DOS}}(\phi, \phi_{\mathrm{R}}) &\equiv 1 + \gamma \cos^2(3(\phi - \phi_{\mathrm{R}})) \end{aligned} \tag{ES15}$$

where λ, β, γ are three coefficients describing the anisotropy (hexagonal wrapping) of each involved physical quantity. One obtains the general isotropic response Λ_{xy}:

$$\Lambda_{xy}(\theta, \phi_{\mathrm{R}}) = \Lambda_{xy}(\theta) = \frac{\pi}{32}\left(3\beta(8 + 18\lambda + 3\gamma(2 + 5\lambda)) + 2(8 + 12\lambda + \gamma(4 + 9\lambda))\right) \sin(\theta) \tag{ES16}$$

independent of the crystallographic orientation ϕ_{R}. This confirms our results of isotropic THz emission. This fulfills the dipolar symmetry for the THz emission *vs.* the in-plane magnetic field direction.

3. *Comparing IREE and ISHE.*

IREE is scaled by the characteristic IREE length Λ_{IEE} whereas the ISHE is scaled by the spin-Hall conductivity (SHC) $\sigma_{\mathrm{SHC}} = \sigma_{xz}^{y}$. In order to compare the efficiency of the two different processes, one must compare Λ_{IEE} to the product $\theta_{\mathrm{SHE}} \lambda_{sf}$ where $\theta_{\mathrm{SHE}} = \sigma_{\mathrm{SHC}} \times \rho_{xx}$ refers to the effective spin-Hall angle and l_{sf} is the spin-diffusion length. One can thus transform Λ_{IEE} to an equivalent SHC according to $\Lambda_{\mathrm{IEE}} = \sigma_{\mathrm{IEE}} \rho_{xx} \times \lambda_{sf} = \sigma_{\mathrm{IEE}} r_s$ with $r_s = \frac{\tau_s}{e^2 \mathcal{N}_{\mathrm{DOS}}^{3\mathrm{D}} \lambda_{sf}}$ the spin resistance.

We arrive at the following formula $\sigma_{\mathrm{IEE}} = \frac{1}{2\sqrt{3}\pi^2}\left(\frac{e^2}{\hbar}\right) k_{\mathrm{F}}^2 \Lambda_{\mathrm{IEE}}$ to compare with σ_{SHE} if non-zero.

B. TB electronic band structure of $Bi_{1-x}Sb_x$.

1. Method.

We have developed a tight binding (TB) model in order to describe the $Bi_{1-x}Sb_x$ electronic band structure as well as their surface topological properties [21]. This approach is indeed well suited for TI and gives a fair description of the surface state spin texture in close agreement with the one derived from Density Functional Theory (DFT) developed for pure Bi surfaces [22–24]. The rhombohedral A7 structure is described by two atoms per unit cell, forming then a bilayer (BL) of thickness of about 0.4 nm. $Bi_{1-x}Sb_x$ slabs are obtained by stacking the BL along the (1 1 1) direction (z axis) with two different plane-to-plane distances. We constructed our Hamiltonian on the basis of the work of Ref. [21] using the generalization of the sp^3 TB-model Hamiltonian proposed for bulk Bi and Sb crystals [25], adapted to $Bi_{1-x}Sb_x$ alloys [26] and complemented by the introduction of additional surface potential terms when dealing with thin layers (treatment in slabs) [21, 27, 28]. In particular, the hopping parameters for the BiSb alloys are obtained by using the virtual crystal approximation (VCA) according to [26]:

$$V_{\mathrm{C}}^{\mathrm{BiSb}} = x\ V_{\mathrm{C}}^{\mathrm{Sb}} + (1-x^2)\ V_{\mathrm{C}}^{\mathrm{Bi}} \tag{ES17}$$

where x is the antimony content and $V_{\mathrm{C}}^{\mathrm{Sb}}$ and $V_{\mathrm{C}}^{\mathrm{Bi}}$ are the respective hopping parameters of Sb and Bi taken from Ref. [25]. One notes $\hat{\sigma}_\alpha$ the spin index on each atom where α stands for the directional index. The hopping terms among the atomic orbitals are decomposed into inter- and intra-BL hopping terms. The inter-BL off-diagonal hopping term between atoms (plane) 1 and atoms (plane) 2 consists of the nearest-neighbor coupling in the bulk BiSb Hamiltonian, whereas the intra-BL hopping term consists of two parts which represents respectively the third and second nearest neighbor contributions. We considered the overall TB Hamiltonian according to:

$$\hat{\mathcal{H}} = \hat{\mathcal{H}}_0 + \hat{\mathcal{H}}_{\mathrm{SO}} + \hat{\mathcal{H}}_\gamma \tag{ES18}$$

where $\hat{\mathcal{H}}_0 = \Sigma_{i\mu}^{j\nu}\, |i\mu\rangle\, V_{i\mu}^{j\nu}\, \langle j\nu|$ represents the hopping Hamiltonian (i, j are the atomic positions, μ, ν are the orbitals), $\hat{\mathcal{H}}_{\mathrm{SO}} = \frac{\hbar}{4m^2c^2}\left(\overrightarrow{\nabla} V(r) \times \hat{p}\right) \cdot \hat{\sigma}$ the spin-orbit term and $\hat{\mathcal{H}}_\gamma$ the equivalent-Rashba surface potential induced by the deformation of the surface orbitals due to the local electric field. Indeed, due to the symmetry breaking at the surface, additional hopping interaction terms

must be taken into account in the Hamiltonian at the two surface planes. We model such effect for the sp^3 basis by using the approach of Ast and Gierz [28] for $\hat{\mathcal{H}}_\gamma$ considering three additional surface hopping terms: on-site γ_{sp_z} hopping, and neighboring hopping terms $\gamma^1_{sp} = \gamma^2_{sp}$ and γ_{pp} acting respectively between the $s - p_z$ and $p_x - p_z$ (or $p_y - p_z$) surface orbitals. We thus add the $\hat{\mathcal{H}}_\gamma$ Hamiltonian term of the form:

$$\hat{\mathcal{H}}_\gamma = \begin{cases} \pm\gamma_{pp}\cos(\theta) & (i,j) \equiv (p_x, p_z) \\ \pm\gamma_{pp}\sin(\theta) & (i,j) \equiv (p_y, p_z) \\ \pm\gamma_{sp} & (i,j) \equiv (s, p_z) \end{cases} \tag{ES19}$$

where the + (-) sign corresponds to the uppermost (lowermost) atomic plane and θ is the angle between the direction joining the two atoms considered and the x-direction. We then restrained ourselves to the in-plane surface hopping as for a pure 2D system. The best agreement with ARPES results is found by adding, as proposed in Ref. [28], additional on-site $s-p_z$ coupling $\gamma_{sp} = -0.2$ eV, and surface hopping terms γ_{sp1}=0.3 eV and γ_{pp}=-0.6 eV for x=0.15 slightly departing from the values given for pure Bi, *i.e.* γ_{sp1}=0.45 eV and γ_{pp}=-0.27 eV [21], with opposite sign for the top and bottom surfaces due to the opposite direction of the potential gradient. We emphasize that this surface terms are required to correctly reproduce the surface state dispersion as observed by ARPES experiments. The size of the Hamiltonian $\hat{\mathcal{H}}(k_x, k_y)$ to diagonalize is $16N \times 16N$ where N is the number of bilayers (BLs). Once the Green function of the multilayer system is defined as:

$$\hat{G}(\varepsilon, k_x, k_y) = \left[\varepsilon + i\delta - \hat{\mathcal{H}}(k_x, k_y)\right]^{-1} \tag{ES20}$$

The partial density of state (DOS) $\mathcal{N}_{\text{DOS}}(\varepsilon)$ *vs.* the energy ε equals $\mathcal{N}(n, \varepsilon) = -(1/\pi)\ \text{Im}\,\text{Tr}[\hat{G}(\varepsilon, n, k_x, k_y)]$ whereas the spin density of states (spin-DOS) with spin along the α direction is $s_\alpha(\varepsilon) = (1/\pi)\ \text{Im}\,\text{Tr}[\hat{\sigma}_\alpha \hat{G}(\varepsilon, k_x, k_y)]$. δ is the typical energy broadening ($\simeq$ 10 meV) and the trace (Tr) is applied over the considered sp^3 orbitals on a given BL index ($n \in [1, N]$). The energy zero (ε=0) refers to the Fermi level position.

2. $Bi_{1-x}Sb_x$ energy dispersion: surface states vs. Bloch states.

Fig. FS15 displays the typical electron energy dispersions obtained for $Bi_{0.79}Sb_{0.21}$ (respectively 2.5 (a), 5 (b) and 15 nm (c)) and corresponding spin-resolved DOS along the $\overline{\Gamma \text{M}}$ direction projected

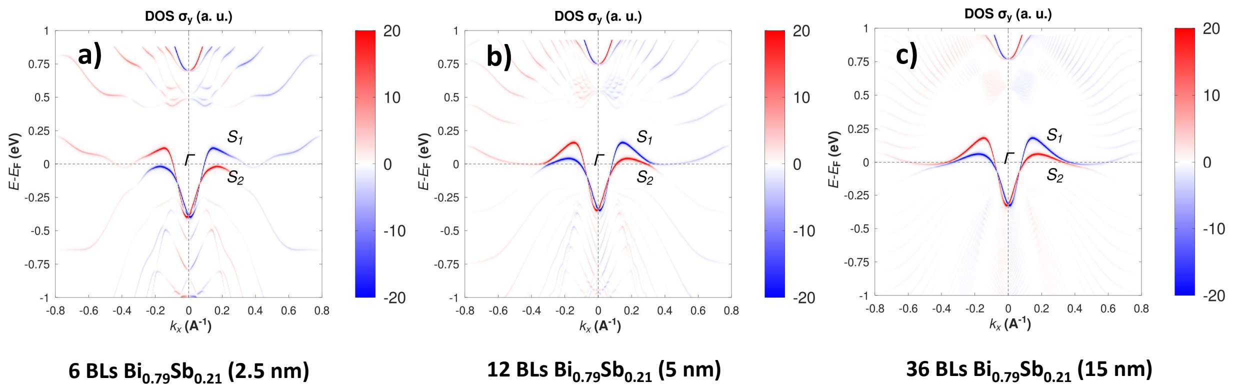

Figure FS15. **$Bi_{0.79}Sb_{0.21}$ energy dispersion as a function of the thickness.** Tight-binding calculations of the dispersion profile of $Bi_{1-x}Sb_x$ as a function of the thickness t_{TI} ranging from (a) 2.5 nm (6 BLs), (b) 5 nm (12 BLs) and (c) 15 nm (36 BLs).

onto the first top BL. This emphasizes on the typical spin-splitting arising on the two S_1 and S_2 surface states. The spin-resolved DOS plotted in colorcode (red for positive value and blue for negative values) represents a large fraction of the total DOS (typically between 50% and 70% corresponding to average typical values $\langle \psi_{\mathbf{k}n} | \sigma_y | \psi_{\mathbf{k}n} \rangle \simeq \pm 0.5 - 0.7\hbar$). The bulk valence and electronic bands are also shown, displaying a specific spin-resolved texture but however much less pronounced.

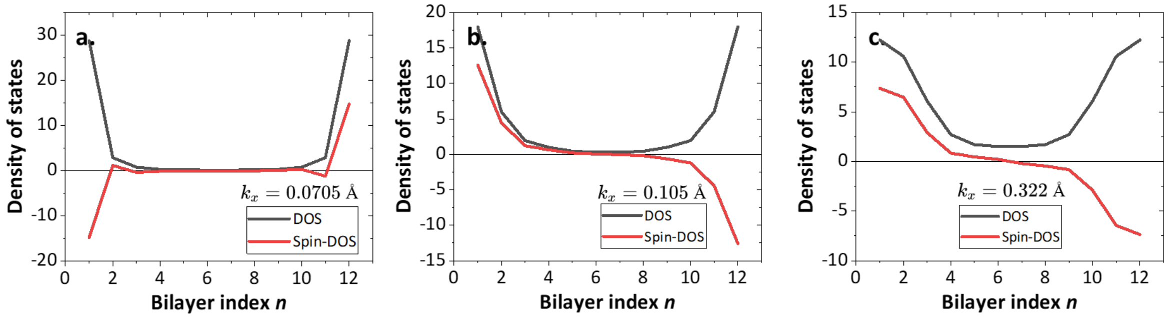

Figure FS16. **Evanescence profile of S_1 TSS and S_2 surface states for calculated for 5 nm $Bi_{0.85}Sb_{0.15}$ (12 BLs).** Values of the density of states (grey) and σ_y-polarized density of states (red) of S_1 TSS in $Bi_{0.85}Sb_{0.15}$(5nm) as a function of the bilayer index n near (a) $k_x = 0.0705$ Å^{-1}, (b) $k_x = 0.105$ Å^{-1} and (c) $k_x = 0.322$ Å^{-1}.

Fig. FS16 displays the typical evanescence of both the S_1 (TSS) and S_2 surface states in depth of the $Bi_{0.85}Sb_{0.15}$ layer. Figs. FS16a,b,c displays both DOS (red) and spin-resolved DOS (red) projected on each BL ($n = 1 - 12$) calculated at the Fermi level ($\varepsilon = 0$) near the $\overline{\Gamma}$ point for $k_{\parallel} = 0.0705$ Å^{-1} (Fig FS16a for S_1), 0.105 Å^{-1} (Fig FS16b for S_2) and 0.322 Å^{-1} (Fig FS16c for S_2 hole pocket) respectively. One note the short decrease of both DOS and spin-resolved DOS at both interfaces of the order of 2 BLs ($\lambda_{evan.} \simeq 0.5$ nm) in the exact range of the results given by Ishida for pure Bi [29]. Note also that the spin-DOS profile is exactly asymmetric from the symmetry point at the center of the layer whereas the DOS profile remains symmetric.

C. TB calculation of the inverse Rashba-Edelstein tensor for $Bi_{1-x}Sb_x$.

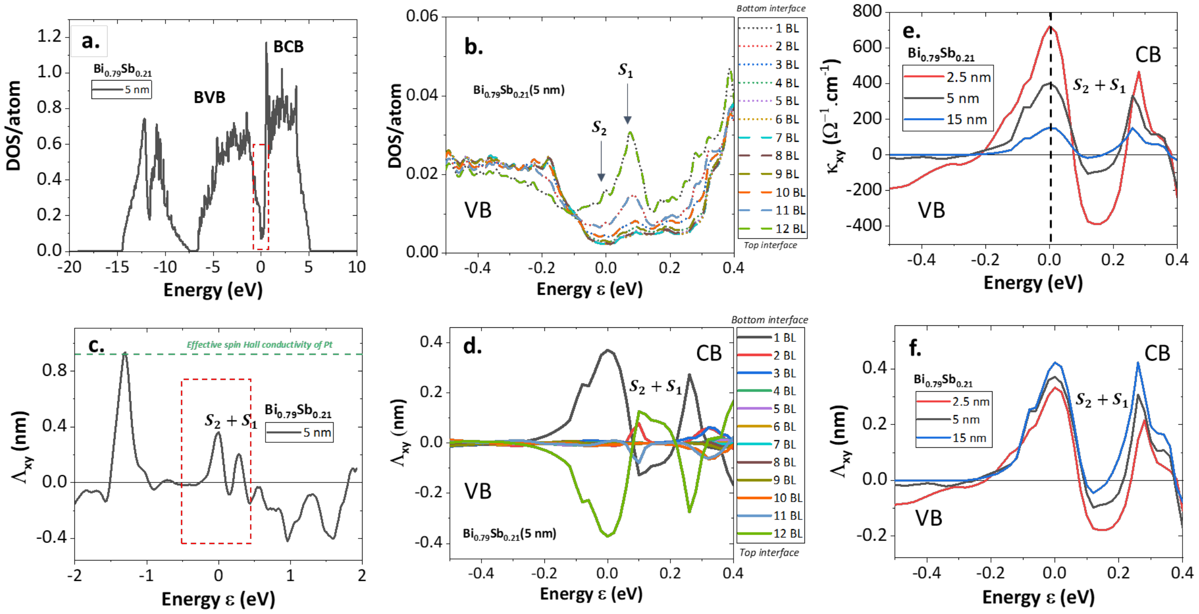

Figure FS17. **TB model and IREE conversion efficiency.** (a) Density of states (DOS) per atom as a function of the energy for $Bi_{0.79}Sb_{0.21}$(5nm)(12 BL). (b) BL-selected DOS in the bandgap region mainly involving the two states S_1 and S_2. (c) IREE length $\Lambda_{xy}^{\mathrm{IREE}}$ calculated in the [-2;2] energy window emphasizing on the S_1 and S_2 contributions in the bandgap. (d) BL-selected contribution to the IREE length $\Lambda_{xy}^{\mathrm{IREE}}$ from the 1st to the 12th bilayer of $Bi_{1-x}Sb_x$(5nm) for $x = 0.21$. (e) IREE tensor κ_{xy} as a function of the $Bi_{0.79}Sb_{0.21}$ thickness (2.5, 5, 15 nm) given in units of the spin Hall conductivity (Ω^{-1}.cm^{-1}). (f) Thickness-renormalized κ_{xy} given in units of $\Lambda_{xy}^{\mathrm{IREE}}$ (nm).

The IREE inverse Edelstein length $\Lambda_{xy}^{\text{IREE}}$ for $Bi_{1-x}Sb_x$ has been calculated within the TB framework. Fig. FS17a reports on typical results obtained for $Bi_{0.79}Sb_{0.21}$(5nm)(12 BLs). Fig. FS17a first displays the density of states (DOS) per atom $\mathcal{N}_{\text{DOS}}(\varepsilon)$ *vs.* the binding/excitation in the -20 to 10 eV window. We clearly observe the presence of the valence (BVB) and conduction bulk bands (BCB) separated by the bandgap marked as dotted red lines where the two surface states S_1 and S_2 lie. We focus here on this region of interest in Fig. FS17b displaying the corresponding DOS per atom for each BL (from the 1st to the 12th) from -0.5 eV to 0.4 eV. One observes that the DOS is not zero in the gap (-0.2 eV - 0.2 eV) owing to the presence of the 4 different SS (S_1 and S_2 TSS at the respective two top and bottom surfaces). By calculating the BL-selective DOS, we can observe an enhancement of the DOS corresponding to the two outwards BLs, labelled by $n = 1$ and $n = 12$, corresponding to the two interfaces. It also proves the strong localization/evanescence of the SS in the depth of the layers as calculated by Ishida by DFT in the case of pure Bi [29]. A significant contribution originates from the first and 12th BL which presents a larger density of states owing to the presence of the two surface states S_1 TSS and S_2.

The IREE length $\Lambda_{xy}^{\text{IREE}}$ is calculated by considering an intraband (spin-) relaxation time of $\tau_s = 10$ fs integrating over the 2D Fermi surface its contribution from the top BL over the mid BiSb thickness where the top evanescent states disappear. We observe in Fig. FS17d that the most important amplitude to the IREE length originates from DOS generated by S_2 and S_1 giving rise to a value of about 0.4 nm at the Fermi level ($\tau_s = 10$ fs). The strong contribution of the IREE response in the bulk valence bands near -1 eV comes from a important spin-texture of surface states or bulk valence bands, which are however hard to probe with our set-up configuration. We present in Fig. FS17e the IREE tensor in the bandgap for different BiSb thickness (respectively 2.5, 5 and 15 nm). We observe that Λ_{xy} follows the same energy dependence and increases on reducing the BiSb thickness. To extract the interfacial IREE figure of merit, we have plotted κ_{xy} integrated over the volume (Fig. FS17e). We recover an almost-independent interconversion profile with the BiSb thickness, which is a strong element for interfacially-mediated interconversion. The extrema of this IREE tensor coincides with the S_2 and S_1 TSS.

In order to go into deeper details, we plot on Fig. FS18b the BL-selected k-dependence of the charge current produced by SCC of a 5 nm $Bi_{0.79}Sb_{0.21}$ film (12 BLs) calculated onto the selected $-\overline{M}\Gamma\overline{\text{M}}$ direction. We have represented the different BL contributions from the first one (1st) receiving the spin-current (from Co) to the one in the middle (6th). The related DOS and

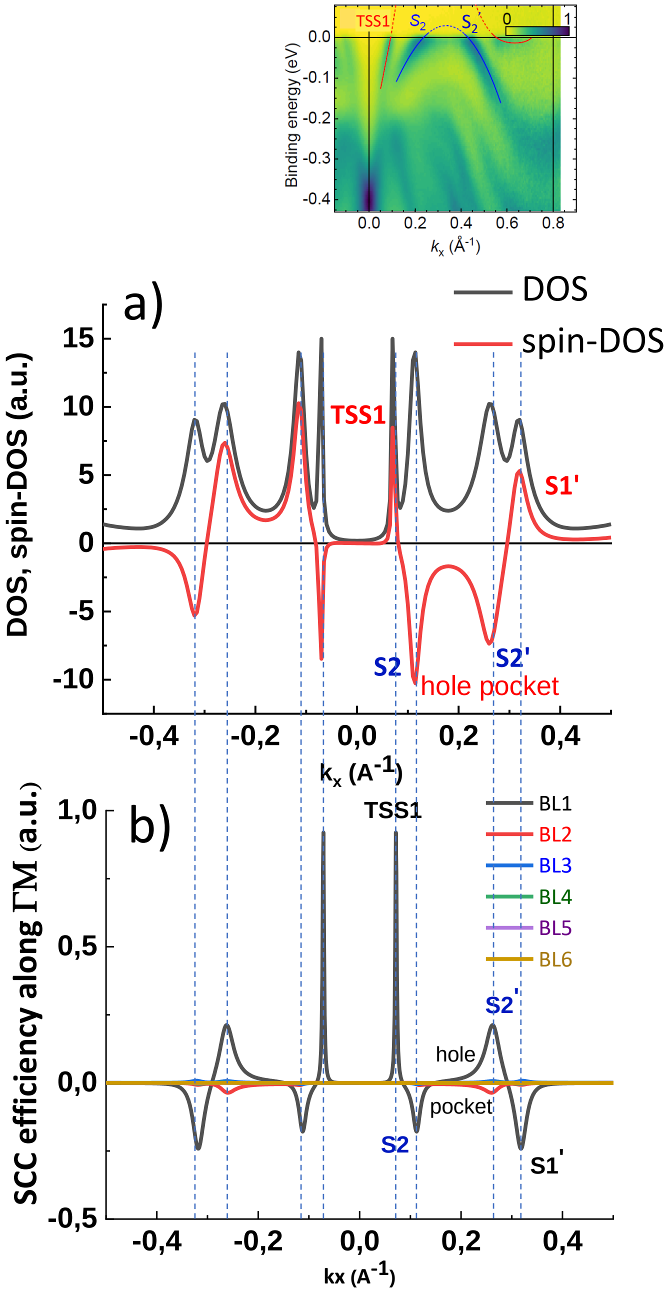

Figure FS18. **Bilayer-selected DOS and signature of the SCC along the -$\overline{M}\overline{\Gamma \mathrm{M}}$ direction calculated on 5 nm thick (12 BLs) $Bi_{0.79}Sb_{0.21}$ calculated at the Fermi level $\varepsilon = 0$ (the 1st BL corresponds to the one at the interface with Co whereas the 6th BL corresponds to the one in the center of the film).** (a) k-dependence of the respective DOS (black) and spin-DOS (red) calculated along k=(-0.5 $\rightarrow$ +0.5 Å^{-1}) along $\overline{\Gamma \mathrm{M}}$ showing the respective topological S_1, S_1' and surface states S_2, S_2' resonances. (b) k-dependence of the SCC (arbitrary units) along $\overline{\Gamma \mathrm{M}}$ showing positive SCC and negative SCC at the corresponding resonances.

spin-resolved DOS are plotted correspondingly on Fig. FS18a. Several resonances on the charge current giving rise to either respective positive (S_1 and S'_2) and negative (S_2 and S'_1) peaks may be observed and gives uncompensated integral contribution. One clearly observes that the major contribution originates only from the 1st bilayer, typically extending over 0.4 nm from the top surface (see Suppl. Mat. S5b), thus explaining the relative thickness insensitivity of the THz signal in the (2.5-15 nm) range, *i.e.* for thin films typically thicker than 1 nm. Even more strikingly, we find that the hole pocket (lying between S_2 and S'_2) giving rise to a positive spin direction (red curve on Fig. FS18a gives rise to a negative (S_2), that is opposite to the one of the S_1 TSS, and a consecutive positive (S'_2) contribution without spin-inversion. This is simply related to the change of the velocity signs of holes from the left part to the hole pocket to the right part of it, as the charge current generated from SCC can be written as $\mathcal{J}_{\text{SCC}} \propto \sigma_y \left(\frac{\partial \mathcal{H}}{\partial k_x}\right)$. The full integration of $\mathcal{J}_{\text{SCC}}$ over the 2D Brillouin zone is displayed on Fig. 3c-d of the manuscript.

[1] L. Baringthon, Élaboration et caractérisation de phases topologiques de l'alliage $Bi_{1-x}Sb_x$ pour la conversion PhD thesis, Université Paris-Saclay, 2022.

[2] L. Baringthon, T. H. Dang, H. Jaffrès, N. Reyren, J.-M. George, M. Morassi, G. Patriarche, A. Lemaitre, F. Bertran, and P. Le Fèvre, "Topological surface states in ultrathin $Bi_{1-x}Sb_x$ layers," Physical Review Materials, vol. 6, p. 074204, July 2022.

[3] T. Seifert, S. Jaiswal, U. Martens, J. Hannegan, L. Braun, P. Maldonado, F. Freimuth, A. Kronenberg, J. Henrizi, I. Radu, E. Beaurepaire, Y. Mokrousov, P. M. Oppeneer, M. Jourdan, G. Jakob, D. Turchinovich, L. M. Hayden, M. Wolf, M. Münzenberg, M. Kläui, and T. Kampfrath, "Efficient metallic spintronic emitters of ultrabroadband terahertz radiation," Nature Photonics, vol. 10, pp. 483–488, Jul 2016.

[4] O. Gueckstock, L. Nádvorník, M. Gradhand, T. S. Seifert, G. Bierhance, R. Rouzegar, M. Wolf, M. Vafaee, J. Cramer, M. A. Syskaki, G. Woltersdorf, I. Mertig, G. Jakob, M. Kläui, and T. Kampfrath, "Terahertz Spin-to-Charge Conversion by Interfacial Skew Scattering in Metallic Bilayers," Advanced Materials, vol. 33, no. 9, p. 2006281, 2021.

[5] M. Meinert, B. Gliniors, O. Gueckstock, T. S. Seifert, L. Liensberger, M. Weiler, S. Wimmer, H. Ebert, and T. Kampfrath, "High-Throughput Techniques for Measuring the Spin Hall Effect," Phys. Rev. Applied, vol. 14, p. 064011, Dec 2020.

[6] J. Hawecker, E. Rongione, A. Markou, S. Krishnia, F. Godel, S. Collin, R. Lebrun, J. Tignon, J. Mangeney, T. Boulier, J.-M. George, C. Felser, H. Jaffrès, and S. Dhillon, "Spintronic THz emitters based on transition metals and semi-metals/Pt multilayers," Applied Physics Letters, vol. 120, no. 12, p. 122406, 2022.

[7] M. Querry, "Optical Constants of Minerals and Other Materials from the Millimeter to the Ultraviolet," in Contractor Report CRDEC-CR-88009, 1987.

[8] E. D. Palik, Handbook of Optical Constants of Solids. Boston: Academic Press, 1998.

[9] W. S. M. Werner, K. Glantschnig, and C. Ambrosch-Draxl, "Optical Constants and Inelastic Electron-Scattering Data for 17 Elemental Metals," Journal of Physical and Chemical Reference Data, vol. 38, no. 4, pp. 1013–1092, 2009.

[10] A. D. Rakić, "Algorithm for the determination of intrinsic optical constants of metal films: application to aluminum," Appl. Opt., vol. 34, pp. 4755–4767, Aug 1995.

[11] H. M. Benia, C. Straßer, K. Kern, and C. R. Ast, “Surface band structure of $Bi_{1-x}Sb_x(111)$,” Physical Review B, vol. 91, p. 161406, Apr. 2015.

[12] B. Lenoir, H. Scherrer, and T. Caillat, “An overview of recent developments for BiSb Alloys,” in Semiconductors and Semimetals, vol. 69, pp. 101–137, Elsevier, 2001.

[13] M. Matthiesen, D. Afanasiev, J. R. Hortensius, T. C. van Thiel, R. Medapalli, E. E. Fullerton, and A. D. Caviglia, “Temperature dependent inverse spin Hall effect in Co/Pt spintronic emitters,” Applied Physics Letters, vol. 116, p. 212405, May 2020.

[14] E. Rongione, S. Fragkos, L. Baringthon, J. Hawecker, E. Xenogiannopoulou, P. Tsipas, C. Song, M. Mičica, J. Mangeney, J. Tignon, T. Boulier, N. Reyren, R. Lebrun, J.-M. George, P. Le Fèvre, S. Dhillon, A. Dimoulas, and H. Jaffrès, “Ultrafast Spin-Charge Conversion at $SnBi_2Te_4$/Co Topological Insulator Interfaces Probed by Terahertz Emission Spectroscopy,” Advanced Optical Materials, vol. 10, no. 7, p. 2102061, 2022.

[15] V. Litvinov, Magnetism in topological insulators. Springer, 2020.

[16] M. P. Marder, Condensed Matter Physics. Wiley, 2000.

[17] K. Shen, G. Vignale, and R. Raimondi, “Microscopic Theory of the Inverse Edelstein Effect,” Phys. Rev. Lett., vol. 112, p. 096601, Mar 2014.

[18] L. Fu, “Hexagonal Warping Effects in the Surface States of the Topological Insulator Bi_2Te_3,” Phys. Rev. Lett., vol. 103, p. 266801, Dec 2009.

[19] S. Vajna, E. Simon, A. Szilva, K. Palotas, B. Ujfalussy, and L. Szunyogh, “Higher-order contributions to the Rashba-Bychkov effect with application to the Bi/Ag(111) surface alloy,” Phys. Rev. B, vol. 85, p. 075404, Feb 2012.

[20] A. Johansson, J. Henk, and I. Mertig, “Theoretical aspects of the Edelstein effect for anisotropic two-dimensional electron gas and topological insulators,” Phys. Rev. B, vol. 93, p. 195440, May 2016.

[21] K. Saito, H. Sawahata, T. Komine, and T. Aono, “Tight-binding theory of surface spin states on bismuth thin films,” Phys. Rev. B, vol. 93, p. 041301, Jan 2016.

[22] Y. M. Koroteev, G. Bihlmayer, J. E. Gayone, E. V. Chulkov, S. Blügel, P. M. Echenique, and P. Hofmann, “Strong Spin-Orbit Splitting on Bi Surfaces,” Phys. Rev. Lett., vol. 93, p. 046403, Jul 2004.

[23] T. Hirahara, T. Nagao, I. Matsuda, G. Bihlmayer, E. V. Chulkov, Y. M. Koroteev, P. M. Echenique, M. Saito, and S. Hasegawa, “Role of Spin-Orbit Coupling and Hybridization Effects

in the Electronic Structure of Ultrathin Bi Films," Phys. Rev. Lett., vol. 97, p. 146803, Oct 2006.

[24] Y. M. Koroteev, G. Bihlmayer, E. V. Chulkov, and S. Blügel, "First-principles investigation of structural and electronic properties of ultrathin Bi films," Phys. Rev. B, vol. 77, p. 045428, Jan 2008.

[25] Y. Liu and R. E. Allen, "Electronic structure of the semimetals Bi and Sb," Phys. Rev. B, vol. 52, pp. 1566–1577, Jul 1995.

[26] J. C. Y. Teo, L. Fu, and C. L. Kane, "Surface states and topological invariants in three-dimensional topological insulators: Application to $Bi_{1-x}Sb_x$," Phys. Rev. B, vol. 78, p. 045426, Jul 2008.

[27] L. Petersen and P. Hedegård, "A simple tight-binding model of spin–orbit splitting of *sp*-derived surface states," Surface Science, vol. 459, no. 1, pp. 49–56, 2000.

[28] C. R. Ast and I. Gierz, "*sp*-band tight-binding model for the Bychkov-Rashba effect in a two-dimensional electron system including nearest-neighbor contributions from an electric field," Phys. Rev. B, vol. 86, p. 085105, Aug 2012.

[29] H. Ishida, "Decay length of surface-state wave functions on Bi(1 1 1)," Journal of Physics: Condensed Matter, vol. 29, p. 015002, nov 2016.
